# Limited tissue persistence confirms attenuation of live-attenuated chikungunya vaccine VLA1553 in non-human primates

**DOI:** 10.1038/s41541-026-01535-4

**Published:** 2026-07-23

**Authors:** Pierre Roques, Andrea Fritzer, Nathalie Dereuddre-Bosquet, Laetitia Bossevot, Quentin Pascal, Roger Le Grand, Urban Lundberg, Andreas Meinke

**Affiliations:** 1 Center for Immunology of Viral, Auto-Immune, Hematological and Bacterial diseases (IMVA-HB/IDMIT), Université Paris-Saclay, INSERM, CEA, Fontenay-aux-Roses, France; 2 Valneva Austria GmbH, Vienna, Austria; 3 Present Address: Institut Pasteur de Guinée, Conakry, Guinea

**Keywords:** Diseases, Immunology, Microbiology

## Abstract

Chronic chikungunya disease is caused by the mosquito-borne chikungunya virus (CHIKV) induced by viral persistence in target tissues. To investigate the risk of viral persistence of the approved live-attenuated vaccine IXCHIQ^®^ (VLA1553), cynomolgus macaques received a 100-fold higher dose than the approved dose for IXCHIQ^®^ or wild-type CHIKV. In VLA1553-treated NHPs, fever was transient, coinciding with peak viremia, with no significant virus detected in tissues except for immune organs. WT CHIKV-treated NHPs developed fever persisting for several days with higher peak viremias, and virus was consistently detected in tissues at higher levels than in VLA1553-treated animals. Additionally, WT CHIKV-treated NHPs showed more CHIKV in saliva and vaginal fluid, lymphocytopenia, increased liver enzymes and plasma prostaglandin E_2_, and higher expression of pro-inflammatory cytokines and chemokines associated with WT CHIKV disease than VLA1553-treated animals. This study demonstrated attenuation of vaccine strain VLA1553, with no critical observations in non-human primates (NHPs) following administration of a high dose.

## Introduction

Chikungunya disease is caused by the chikungunya virus (CHIKV), which is typically transmitted by *Aedes aegypti* and *Aedes albopictus* mosquitoes and is endemic throughout Africa, Asia, the Americas, and parts of Europe. Following CHIKV infection, acute symptoms can include high fever, polyarthralgia, and intense myalgia^1–5^, and approximately 43% of infected individuals will subsequently experience longer-term, debilitating, chronic symptoms including arthralgia that may continue for months or years^4,6–9^. In the future, both *Aedes aegypti* and *Aedes albopictus* are expected to continue expanding further into temperate zones, including Europe and the US, through increasing international travel and the spread of potential mosquito vectors through global warming and intensification of trade^10,11^. As such, CHIKV is considered a serious threat to global health and has been designated a priority pathogen by governmental and international health organizations around the globe (https://www.niaid.nih.gov/research/emerging-infectious-diseases-pathogens, https://cdn.who.int/media/docs/default-source/consultation-rdb/prioritization-pathogens-v6final.pdf?sfvrsn=c98effa7_9&download=true, https://cepi.net/priority-pathogens). In the absence of any antiviral treatment for chikungunya disease, addressing this substantial unmet medical need, Valneva developed the licensed, live-attenuated vaccine IXCHIQ^®^ (VLA1553) available for active immunization against CHIKV infection^12–15^. The vaccine is derived from the La Réunion isolate LR2006‑OPY1, belonging to the East/Central/South African (ECSA) genotype^16^. The virus attenuation is the result of a 61-amino acid deletion in the C‑terminal region of the non‑structural protein 3 (nsP3), a multifunctional protein essential for CHIKV replication and pathogenesis^17–19^. Prior to the clinical characterization of VLA1553, preclinical studies were conducted to characterize the attenuation of VLA1553 in non-human primates (NHPs) as NHPs were previously shown to be susceptible to CHIKV infection and to reproduce the viral, clinical, and pathological features observed in humans^20,21^. When cynomolgus macaques (*Macaca fascicularis)* were infected with wild type (WT) CHIKV strain LR2006-OPY1, the parental strain of VLA1553, via the intravenous or intradermal route using a viral dose similar to the dose secreted from mosquito salivary glands WT CHIKV was shown to target the lymphoid tissues, liver, central nervous system, joints, and muscles, infecting mainly macrophages, dendritic cells, and some endothelial cells during the acute phase of infection^21^. In the chronic phase of infection, WT CHIKV persisted in the lymphoid organs, liver, joints, and muscles. A single vaccination with VLA1553 via the subcutaneous route at a dose of 10^5^ PFU was previously demonstrated to generate high titers of neutralizing antibodies that persisted for months after immunization and conferred protection from wild-type CHIKV challenge^22^. VLA1553 proved to be safe, and the vaccinated animals did not show any CHIKV-associated findings such as fever, lymphopenia, rash, or joint swelling, in contrast to wild-type CHIKV-infected animals. Antibody responses induced by VLA1553 showed cross-neutralization, as VLA1553, based on an ECSA-IOL strain, generated neutralizing antibodies against a Caribbean strain of CHIKV. Furthermore, in a passive transfer study conducted in cynomolgous macaques, a surrogate of protection for VLA1553 was determined at a µPRNT_50_ titer ≥150 based on protection from viremia (lack of detectable CHIKV RNA) and the absence fever^23^.

The aim of this study was to investigate whether the attenuation of VLA1553 reduces or eliminates the persistence observed for WT CHIKV. We inoculated the same NHP species previously studied (i.e., *Macaca fascicularis*) with VLA1553 or WT CHIKV via the intramuscular route, the route used for human vaccine administration, and at a 2 log higher dose than the approved dose of IXCHIQ^®^. Following inoculation with VLA1553 or WT CHIKV, we evaluated fever and the presence of CHIKV in NHP plasma, body fluids, and tissues, as well as hematological and biochemical parameters, cytokines/chemokines, and histology.

## Results

### Study design

**Fig. 1:**
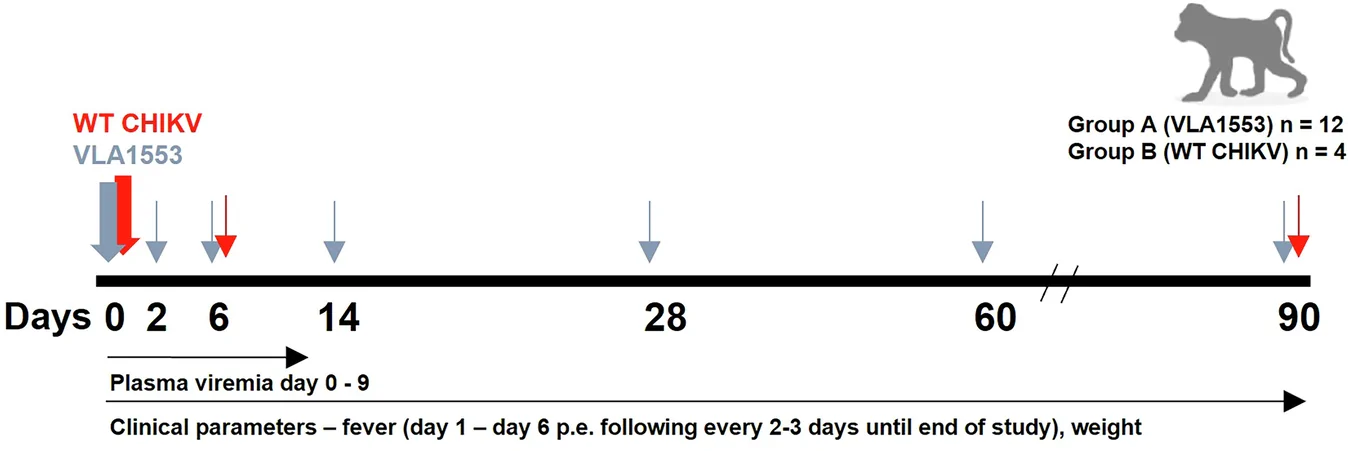
Study design. NHPs were inoculated with VLA1553 (*N* = 12) or WT CHIKV (*N* = 4) on Day 0. VLA1553-exposed animals (2 per time point) were sequentially euthanized on Days 2, 6, 14, 28, 60, and 90 post-exposure, and WT CHIKV-exposed animals (2 per time point) were necropsied on Days 6 and 90. Evaluations included plasma viremia, fever, body weight, complete blood counts, viral shedding or CHIKV presence in NHP body fluids and in various tissues, blood biochemistry, signs of inflammation typically associated with WT CHIKV infection, and histopathological examination of selected organs. At the following time points, body fluids were analyzed: saliva and vaginal fluid on days 2, 4, 6, 60, and 90 for WT CHIKV-exposed animals; for VLA1553-exposed animals, saliva was analyzed on Days 2, 4, 6, 9, 13, 30, 60, and 90 and vaginal fluid on days 2, 4, 6, 30, 60, and 90. Urine, synovial, and cerebrospinal fluid were collected and analyzed at euthanasia.

We assessed tissue dissemination and persistence of CHIKV in NHPs (cynomolgus macaques) using VLA1553 in comparison to the WT CHIKV LR2006-OPY1 administered at a dose that was 2 logs higher than that used in Phase 3 clinical development^24,25^. Cynomolgus macaques were chosen since CHIKV in this NHP has been previously shown to provide a good model for the human condition^21^. The study included 16 animals, of which 12 NHPs (*N* = 12) received intramuscular 3.2 × 10^6^ TCID_50_ VLA1553 in the extensor digitalis communis muscle and 4 NHPs (*N* = 4) received the corresponding WT CHIKV LR2006-OPY1 at the same dose and route. Inoculation was performed on Day 0. VLA1553-exposed animals (2 per time point) were then sequentially euthanized on Days 2, 6, 14, 28, 60, and 90 post-exposure, and WT CHIKV-exposed animals (2 per time point) were necropsied on Days 6 and 90 post-exposure (Fig. 1). The following parameters were assessed: plasma viremia, fever, and body weight (clinical parameters), complete blood counts, viral shedding or CHIKV presence in NHP body fluids and in various tissues. Furthermore, blood biochemistry and signs of inflammation that are typically associated with WT CHIKV infection were monitored, and a histopathological examination of selected organs was performed.

### Significantly reduced plasma viremia in VLA1553-exposed NHPs

**Fig. 2:**
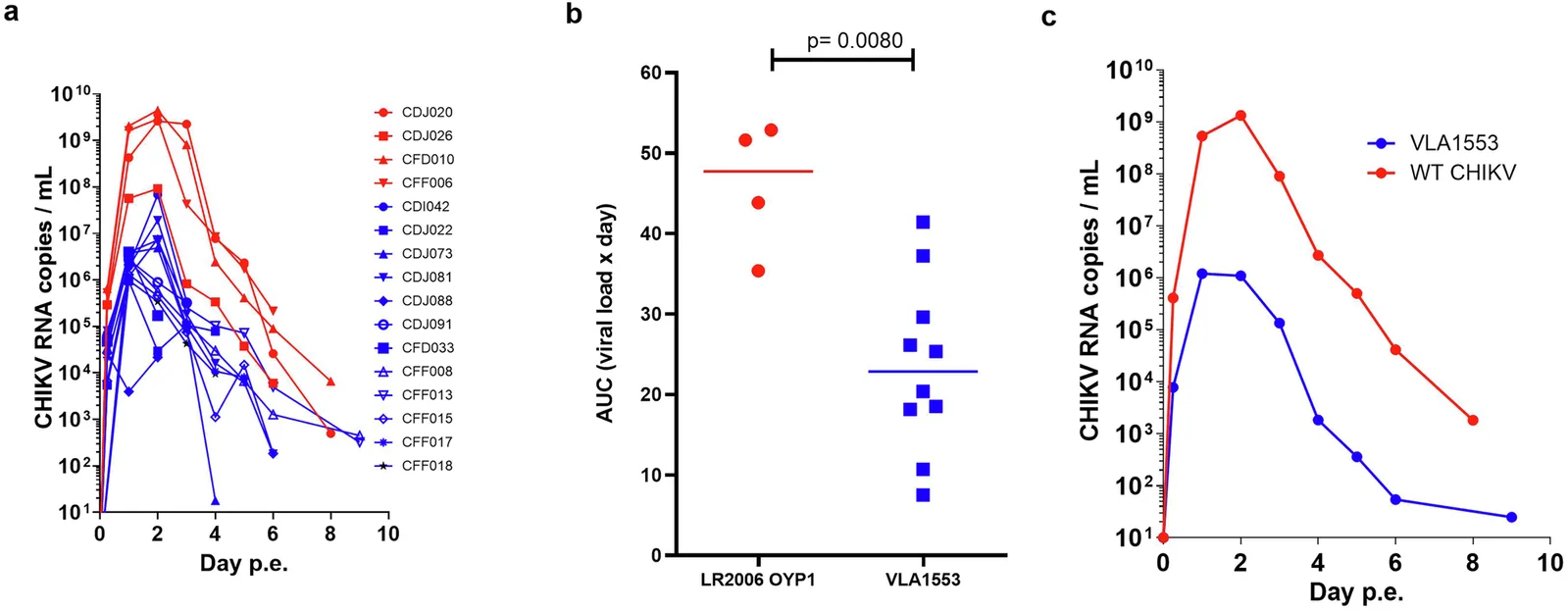
CHIKV titer in NHP plasma determined by RT-qPCR. Data are presented for VLA1553-exposed NHPs (in blue) and WT CHIKV-exposed NHPs (in red) for **a** Individual kinetics of CHIKV viral load (RNA copies/mL) (**b**) AUC for CHIKV RNA copies/mL versus time curves. **c** Geometric mean titers (CHIKV RNA copies/mL). Plasma viral load from Day 0 to Day 4 was determined by classical RNA extraction and RT-qPCR with a LLOQ of 5000 RNA copies/mL (62.5 copies/reaction) and a LOD_95%_ of 720 RNA copies/mL (9 copies/reaction). Plasma viral load below 300 copies/mL in classical RT-qPCR and samples from Day 5 to Day 9 was determined by ultrasensitive RNA extraction and RT-qPCR with a LLOQ of 500 RNA copies/mL (42 copies/reaction) and a LOD_95%_ of 60 RNA copies/mL (5 copies/reaction). Negative values were imputed to 10 RNA copies/mL. For statistical analyses (**b**), the Mann–Whitney test was used.

CHIKV viremia after exposure of NHPs to VLA1553 or WT CHIKV was assessed in plasma as early as 6 hours post-exposure over a period of 9 days. CHIKV viremia in humans, although not widely reported, generally occurs soon after exposure and lasts less than 1 week^26–28^. We detected CHIKV RNA in plasma at 6 hours after inoculation in 12/14 animals in VLA1553 exposed animals and in all animals receiving WT CHIKV (Fig. 2a). Statistical analysis of response kinetics showed a significantly lower AUC (area under the curve) for VLA1553 than WT CHIKV (*p* = 0.0080) (Fig. 2b). In both groups, mean peak viral production was observed on Day 2, with the magnitude being approximately 3 logs lower for VLA1553 than for WT CHIKV-exposed animals (Fig. 2c). The geometric mean (95% confidence interval [CI]) peak titers were significantly lower in VLA1553-treated animals than WT CHIKV-exposed animals (2.9 × 10^6^ [8.4 × 10^5^–9.8 × 10^6^] RNA copies/mL versus 1.3 × 10^9^ [7.6 × 10^7^–2.3 × 10^10^] RNA copies/mL; *P* < 0.01, Mann–Whitney test). The means (95% CIs) were 1.0 × 10^7^ [2.1 × 10^6^–2.2 × 10^7^] RNA copies/mL for VLA1553-exposed animals and 2.5 × 10^9^ [3.7 × 10^8^–5.4 × 10^9^] RNA copies/mL for WT CHIKV-exposed animals.

### Attenuation of VLA1553 leads to reduced clinical observations

**Fig. 3:**
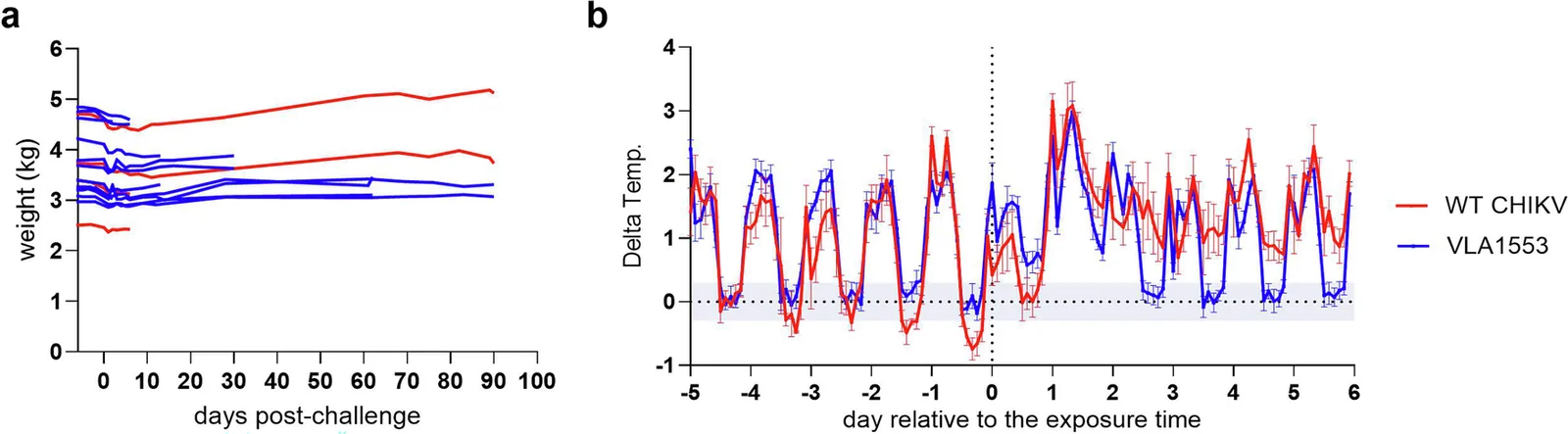
Body temperature and weight measured in NHPs exposed to WT CHIKV and VLA1553. Data are presented for VLA1553-exposed NHPs (in blue) and WT CHIKV-exposed NHPs (in red) for (**a**) body weight which was determined at baseline and daily from the day of viral exposure until Day 6 and every 2–3 days thereafter and (**b**) body temperature which was monitored with implanted STAR ODDI chips every 2 hours. Temperature was normalized to the night temperature (baseline on 10 consecutive nights (10 PM–6 AM). Temperature drops due to anesthesia (around 10 AM) were removed for clarity. Remaining drops in the middle of the day were due to nap periods. Eight animals are presented in the VLA1553 group, as two were euthanized on day 2, and there were 2 defective chips reported.

As part of clinical observations, body weight and body temperature were assessed while symptoms of CHIKV infection, such as arthralgia, headache, and myalgia, which have been documented in humans infected with CHIKV, were not reported in our study due to the challenges of measuring these in an animal model. Body weight was determined at baseline and daily from the day of VLA1553 or WT CHIKV exposure until Day 6 and every 2–3 days thereafter. In both VLA1553- and WT CHIKV-exposed animals, a small drop in the animals’ weights was observed on Day 1 post-inoculation, most probably related to the virus inoculation, which returned back to baseline levels by Day 2 (Fig. 3a). No significant decreases in weight were detected in WT CHIKV- and VLA1553-exposed animals. Body temperature was measured every 2 hours after viral exposure using chips that were implanted subcutaneously in the upper back. In VLA1553-exposed animals, fever was only observed transiently on Day 1 and Day 2 during peak viremia, in contrast to WT CHIKV-exposed animals that experienced fever from Day 1 to Day 6, which peaked on Day 1 and Day 2, coinciding with peak viremia (Fig. 3b).

### VLA1553 minimally and transiently present in body fluids

**Fig. 4:**
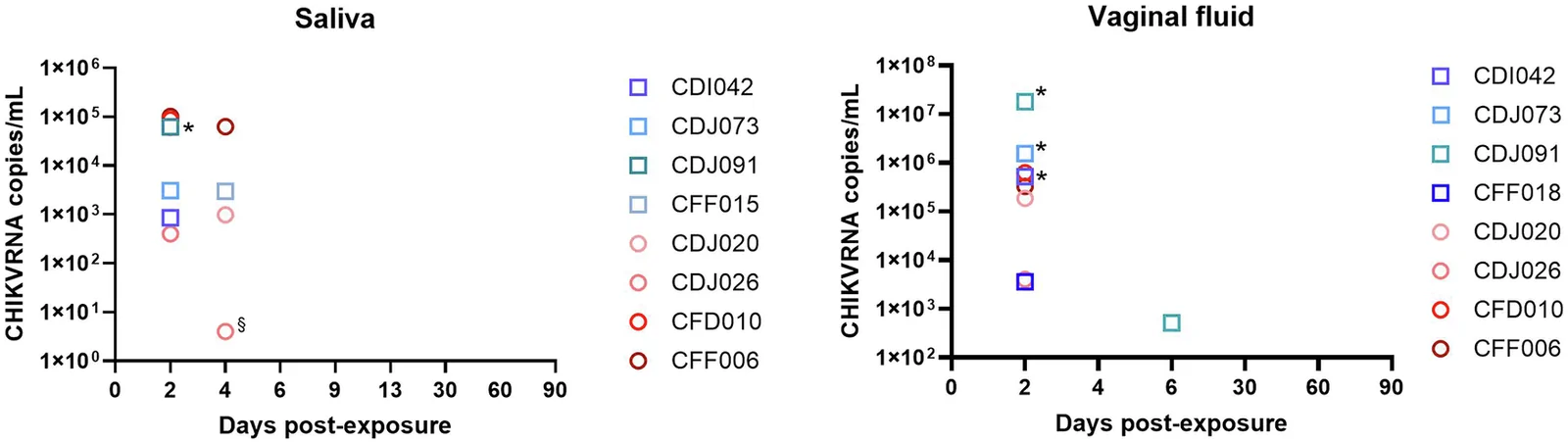
CHIKV RNA detection in saliva and vaginal fluid. Saliva and vaginal fluid were analyzed in WT CHIKV-exposed animals on days 2, 4, 6, 60, and 90 and in VLA1553-exposed animals on days 2, 4, 6, 9, 13, 30, 60, and 90 and days 2, 4, 6, 30, 60, and 90, respectively. Red round symbols represent WT CHIKV-exposed animals, blue square symbols represent VLA1553-exposed animals (different shading representative for different animals). No saliva could be retrieved from Day 4 for the 4th WT CHIKV-exposed animal in repetitive trials due to samples containing too many contaminants (food remains). (*) VLA1553 exposed animals with saliva and vaginal fluid contaminated by blood due to gingival bleeding and menstruation, respectively. (&) Just below LLOQ (500 copies/mL) of ultrasensitive RT-qPCR.

To assess CHIKV in body fluids after exposure to VLA1553 and WT CHIKV and to collect information on whether VLA1553, in comparison to WT CHIKV, could be released into the environment, viral shedding and presence were assessed in NHP saliva and urine, as well as in vaginal, synovial, and cerebrospinal fluid. Following exposure, WT CHIKV showed consistent and detectable shedding in saliva in all animals up to Day 4 (Fig.4). In contrast, VLA1553 demonstrated minimal evidence of shedding: CHIKV RNA was detected in only 3 of 12 animals, limited to Day 2 post‑exposure. Among these, one apparent high viral signal was likely attributable to blood contamination rather than true viral shedding, e.g., due to gingival bleeding, which has been observed in previous studies in NHPs^21^ as well as in CHIKV-infected humans^29,30^. By Day 4, only a single VLA1553‑exposed animal was RNA‑positive, and no CHIKV RNA (WT CHIKV or VLA1553-specific RNA) was detected beyond Day 4. Overall, VLA1553 exhibited markedly reduced presence in body fluids compared with WT CHIKV, with no evidence of sustained replication or prolonged shedding beyond Day 4.

In vaginal fluid, WT CHIKV was consistently detected in all animals on Day 2 post‑exposure (Fig. 4). In contrast, VLA1553 was only observed in 4 of 12 animals, three of which were likely influenced by blood contamination due to menstruation. No CHIKV RNA was detected in cerebrospinal fluid, synovial fluid, or urine in either group, indicating absence of viral dissemination to these compartments.

### VLA1553 shows reduced, transient, and more sporadic tissue distribution compared to WT CHIKV

To evaluate viral load in the main CHIKV target tissues, 2 animals exposed to VLA1553 were sacrificed at each time point (Days 2, 6, 14, 28, 60, and 90), and 2 animals exposed to WT CHIKV were sacrificed at Day 6 and Day 90. We assessed both CHIKV genomic RNA relative to the endogenous control GAPDH using RT-qPCR (reporting ratios of CHIKV RNA to cellular GAPDH RNA) and replicating CHIKV (for selected tissues only) in homogenized tissue lysates using TCID_50_ assay.

#### Spleen and lymph nodes

**Fig. 5:**
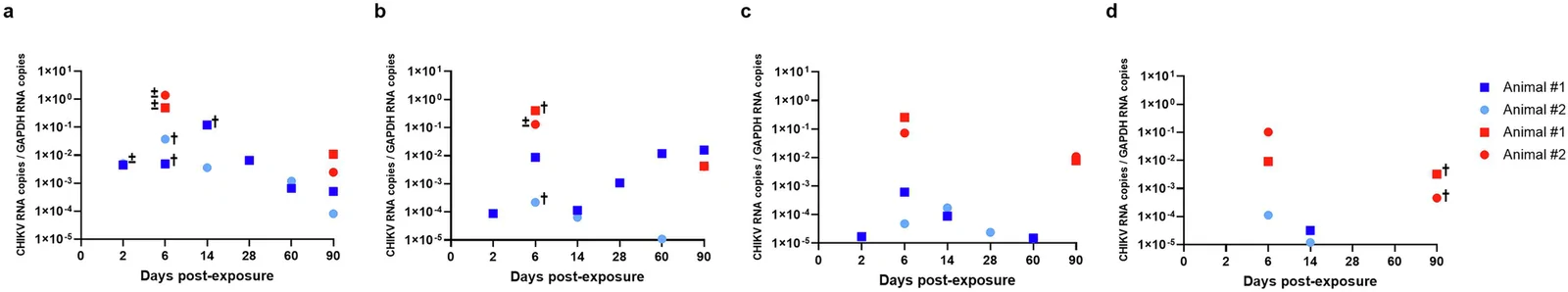
CHIKV/GAPDH RNA ratio in lymphoid tissues. **a** Spleen, (**b**) Axillary, (**c**) Inguinal, and (**d**) Nasopharyngeal lymph nodes were analyzed on Day 6 and Day 90 in WT CHIKV-exposed NHPs (in red) and on Days 2, 6, 14, 28, 60, and 90 in VLA1553-exposed NHPs (in blue) (2 animals were sacrificed in each group per time point). Each value is the mean of 4 measurements, two extracted samples per tissue, performed in duplicates in RT-qPCR. Relative copy number for each sample was calculated with the following equation: Relative copy number = 2-(Cq CHIKV–Cq GAPDH). ±Replicating WT CHIKV or VLA1553 were detected in a TCID_50_ assay. †No replicating WT CHIKV or VLA1553 was detected when assessed in the TCID_50_ assay.

CHIKV RNA was detected in the spleen in both groups; however, levels in VLA1553‑exposed animals were approximately 2 logs lower than in WT CHIKV-exposed animals, with one of 2 VLA1553-exposed animals falling below the limit of detection by Day 28 post-exposure (Supplementary Table 1 and Fig. 5a). Both WT CHIKV and VLA1553 RNA were detectable on Day 90 post-exposure, with lower levels for VLA1553. Across lymph nodes, VLA1553 consistently showed lower RNA levels compared to WT CHIKV, with reductions of 2–3 logs in axillary and inguinal lymph nodes and up to 4 logs in nasopharyngeal lymph nodes (Supplementary Table 1 and Figs. 5b–d).

In axillary lymph nodes, i.e., the draining lymph nodes of the virus inoculation site, VLA1553 was consistently detectable in 2 of 2 animals on Day 6, 14 and 60 (Supplementary Table 1 and Fig. 5b), whereas on Day 2, 28 and 90 VLA1553 RNA was detected in one animal only (Supplementary Table 1 and Fig. 5b). However, WT CHIKV showed substantially higher levels (with a CHIKV/GAPDH ratio that was 2–3 logs higher than for VLA1553) early after exposure on Day 6 before declining to levels comparable to VLA1553 by Day 90. In inguinal lymph nodes, which are distant to the inoculation site of the virus, VLA1553 RNA remained detectable up to Day 60 but at consistently lower levels on Day 6 post-exposure (CHIKV/GAPDH ratio 2–3 logs lower) than for WT CHIKV and was no longer detectable by Day 90, whereas WT CHIKV persisted (Supplementary Table 1 and Fig. 5c). In nasopharyngeal lymph nodes, which are associated with saliva, VLA1553 RNA was only rarely detected with one animal on Day 6 and both animals on Day 14—for both animals at low levels, while WT CHIKV RNA persisted through Day 90 (Supplementary Table 1 and Fig. 5d). Overall, VLA1553 demonstrated reduced magnitude in comparison to WT CHIKV but RNA was detectable until Day 90 in spleen and axillary lymph nodes.

#### Liver

**Fig. 6:**
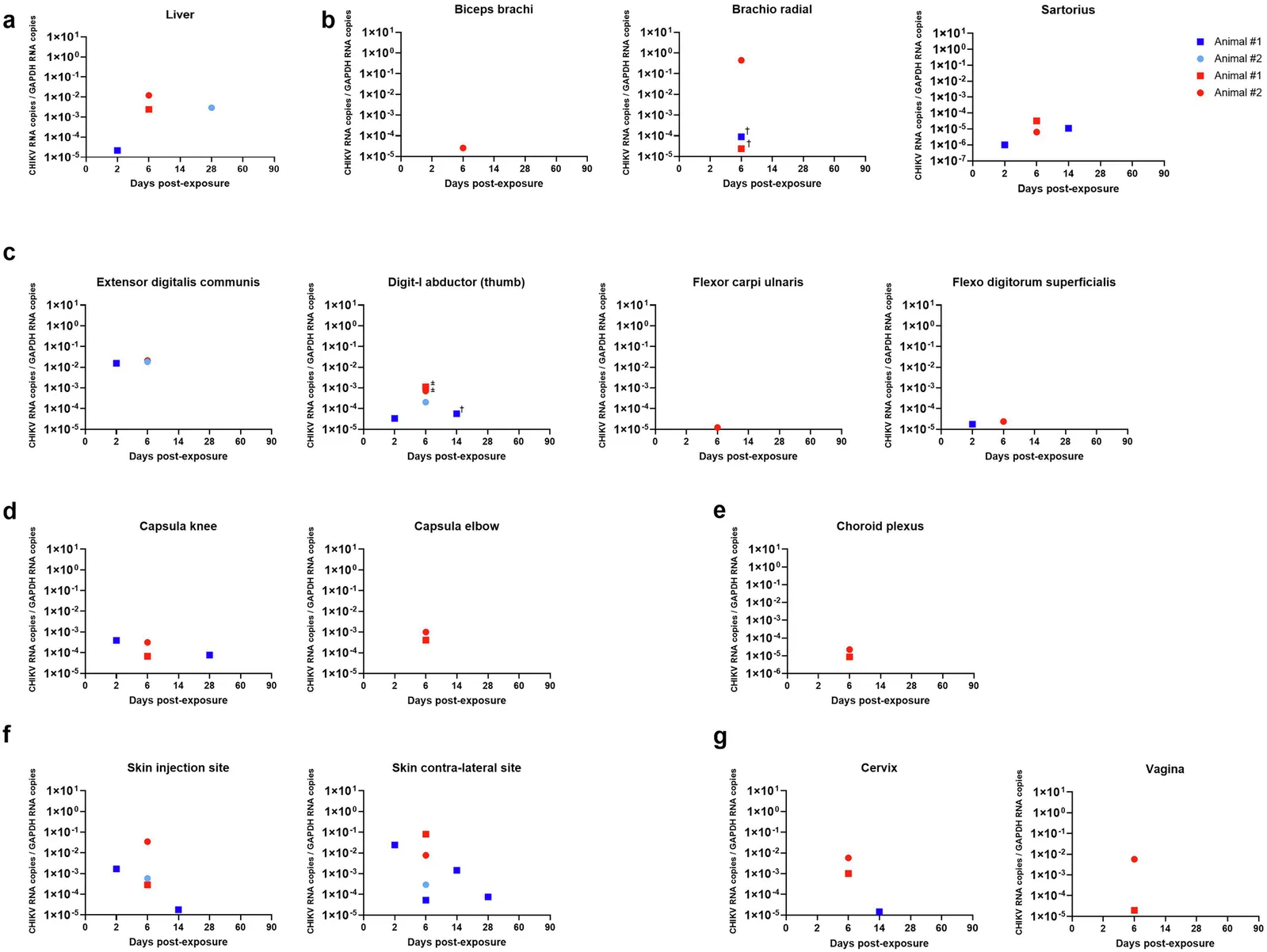
CHIKV/GAPDH RNA ratio in NHP tissues. Tissue viral loads after WT CHIKV and VLA1553 exposure of (**a**) liver, (**b**) arm muscles, (**c**) hand and finger muscles, (**d**) joints, (**e**) brain, (**f**) skin, and (**g**) reproductive tract. Tissues were analyzed on Day 6 and Day 90 in WT CHIKV-exposed NHPs (in red) and on Days 2, 6, 14, 28, 60, and 90 in VLA1553-exposed NHPs (in blue) (2 animals were sacrificed in each group per time point). Each value is the mean of 4 measurements, two extracted samples per tissue performed in duplicates in RT-qPCR, except for liver samples in VLA1553-exposed animals, where 3 extractions per RT-qPCR were performed. Relative copy number for each sample was calculated with the following equation: Relative copy number = 2-(Cq CHIKV–Cq GAPDH). ±Replicating WT CHIKV or VLA1553 were detected in a TCID50 assay. †No replicating WT CHIKV or VLA1553 was detected when assessed in the TCID50 assay.

VLA1553 RNA was largely undetectable, with only low RNA levels observed in two animals (on Day 2 in 1 animal (2.15 × 10^−5^) and on Day 28 in 1 animal (2.96 × 10^−3^), Fig. 6a). In contrast, WT CHIKV produced higher RNA levels, detected in both animals at Day 6. WT CHIKV and VLA1553 were not detected on Day 90 post-exposure.

#### Arm and hand/finger muscles

The presence of CHIKV RNA was assessed in the arm muscles (biceps brachi and brachio radial from the right arm, and sartorius from the left arm, Fig. 6b) as well as in the hand/finger muscles (digit-I abductor [thumb], extensor digitalis communis, flexor carpi ulnaris, flexor digitorum profundus, and flexor digit superficialis, Fig. 6c). VLA1553 RNA was detected only sporadically and at generally low levels across muscle tissues, with inconsistent findings limited to individual animals and timepoints. VLA1553 RNA was detected in the brachio-radial muscle at Day 6 in 1 of 2 animals (8.96 × 10^−5^) in the sartorius muscle on Day 2 and Day 14 in 1 of 2 animals (1.03 × 10^−6^ and 1.11 × 10^−5^, respectively, Fig. 6b). In the extensor digitalis communis muscle VLA1553 was detected on Day 2 in 1 animal (1.55 × 10^−2^) and on Day 6 in 1 animal (1.82 × 10^−2^), in the digit-l abductor muscle on Day 2, Day 6, and Day 14 in 1 animal (3.36 × 10^−5^, 2.05 × 10^−4^ and 5.64 × 10^−5^, respectively), and in the flexor digitorum superficialis on Day 2 in 1 of 2 animals (1.76 × 10^−5^, Fig. 6c). Several muscles, including the biceps brachii and flexor carpi ulnaris, remained consistently negative. In contrast, WT CHIKV RNA was more consistently detected across multiple arm and hand/finger muscles at Day 6, including both proximal and distal sites, although levels remained low overall—in the biceps brachi (1 of 2 animals, 2.60 × 10^−5^), brachio-radial (2 of 2 animals, 2.39 × 10^−5^ and 4.44 × 10^−1^), sartorius (2 of 2 animals, 3.26 × 10^−5^ and 6.42 × 10^−6^), extensor digitalis communis (1 of 2 animals, 2.09 × 10^−2^), digit-l abductor (2 of 2 animals, 1.11 × 10^−3^ and 7.14 × 10^−4^), and flexor carpi ulnaris (1 of 2 animals, 1.23 × 10^−5^) muscles (Fig. 6b, c). Neither VLA1553 nor WT CHIKV RNA was detected in the flexor digitorum profundus at any time point. Overall, VLA1553 demonstrated limited, transient, and less consistent muscle tissue presence.

#### Capsular knee joint and capsular elbow joint

VLA1553 RNA was minimally detected in joint tissues, with low‑level signals observed only in the knee joint of a single animal at isolated timepoints at Day 2 (3.92 × 10^−4^) and at Day 28 (7.73 × 10^−5^) and no detection in the capsula elbow joint (Fig. 6d). In comparison, in WT CHIKV-exposed NHPs, CHIKV RNA was detected in both joints in 2 of 2 animals on Day 6 (capsula knee joint: 6.71 × 10^−5^ and 3.13 × 10^−4^; capsula elbow joint: 4.10 × 10^−4^ and 1.00 × 10^−3^), but was no longer detected in either joint at Day 90 (Fig. 6d).

#### Brain

No CHIKV RNA (neither VLA1553 nor WT CHIKV) was detected in the cerebellum, frontal encephalon, and mesencephalon. However, in both WT CHIKV-exposed animals, viral RNA was detected in the choroid plexus at Day 6 (8.63 × 10^−6^ and 2.23 × 10^−5^) but not at Day 90, and not in any VLA1553-exposed animal at any time point (Fig. 6e).

#### Skin

Both VLA1553 and WT CHIKV RNA were detected at the injection and contralateral sites (Fig. 6f). In animals exposed to VLA1553, RNA was intermittently detected at both sites and declined progressively - RNA was detected on Day 2, Day 6 and Day 14 in 1 animal (1.68 × 10^−3^, 5.88 × 10^−4^ and 1.78 × 10^−5^, respectively), and at the contralateral site on Day 2, 14, and 28 for 1 of the 2 animals (2.45 × 10^−2^, 1.46 × 10^−3^, and 7.33 × 10^−5^, respectively) and on Day 6 in both animals (5.28 × 10^−5^, 2.94 × 10^−4^). WT CHIKV RNA was consistently detected in both animals at both sites on Day 6, with higher levels than VLA1553 at the contralateral site. All values were below the limit of detection at Day 90.

#### Reproductive tissues

VLA1553 RNA was essentially absent from reproductive tissues, with only a single low-level detection in the cervix of one animal on Day 14 post-exposure ([1.45 × 10^−5^], Fig. 6g). In contrast, WT CHIKV RNA was consistently detected in both cervix and vagina in all animals at Day 6, but was no longer detectable by Day 90.

### VLA1553 shows minimal presence of replicating virus

As RT-qPCR analyses determined total CHIKV RNA (including RNA derived from non-viable and viable CHIKV particles), tissues with reasonably high RNA copies detected were selected for a TCID_50_ assay with a pre-amplification step on mosquito cells to assess the presence of live and replicating CHIKV. The advantage of using the TCID_50_ assay with a pre-amplification step on C6/36 cells was that even very low amounts of replicating CHIKV could be detected. However, quantification (as in the Vero cell-based TCID_50_ assay) was not possible due to the pre-amplification step, and so it was only possible to determine whether replicating CHIKV was present or absent (below the limit of detection) in the tested tissues. Replicating VLA1553 was detected only once, in the spleen of a single animal at Day 2 during peak viremia (Fig. 5a). In contrast, replicating WT CHIKV was consistently detected at Day 6, i.e., at the time of a lower viral load after peak viremia across multiple tissues, including the spleen (both animals, Fig. 5a), axillary lymph node (in one of 2 animals, Fig. 5b), and muscle (digit abductor in both animals, Fig. 6c). No replicating virus was detected in other evaluated tissues—the muscle brachio, nasopharyngeal lymph node, mesencephalon and cerebellum—for either group. Overall, VLA1553 showed minimal evidence of replication.

### Limited hematologic effects following VLA1553 exposure

**Fig. 7:**
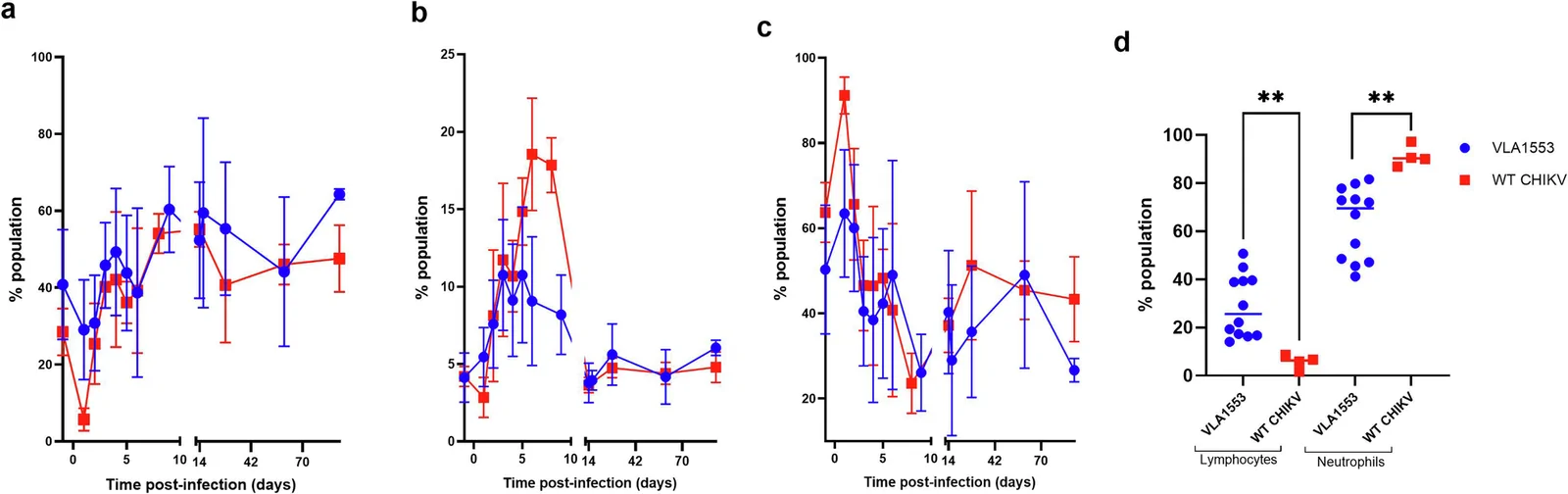
Mean values of percentages of lymphocytes, monocytes, and neutrophils within white blood cells. Data are presented for VLA1553-exposed NHPs (in blue) and WT CHIKV-exposed NHPs (in red) for (**a**) lymphocytes, (**b**) monocytes, (**c**) neutrophils, (**d**) comparison of the maximum decrease in lymphocytes and maximum increase in neutrophils observed at Day 1 post-CHIKV exposure. Baseline values are shown as Day −1. No significant differences were observed for lymphocytes, monocytes, or neutrophils for animals exposed to VLA1553 (Wilcoxon rank test, *p* > 0.05). The maximum decrease in lymphocytes and maximum increase in neutrophils were significantly different between animals exposed to VLA1553 and WT CHIKV (Mann–Whitney test; **p < 0.01).

WT CHIKV infections have previously been described to be associated with increased levels of monocytes or lymphopenia^22^, and therefore, during the follow-up, animals were also monitored for hematological parameters. VLA1553 exposure did not result in statistically significant changes (*p* > 0.05, Wilcoxon rank test) in hematologic parameters for lymphocytes, monocytes, neutrophils, basophils, eosinophils, hemoglobin, platelets, and red blood cells, despite the very high dose administered, with no statistically significant difference from baseline (Fig. 7 and Supplementary Fig. 1). In contrast, for WT CHIKV-exposed animals, however, there was a large decrease in lymphocytes and a large increase in neutrophils after inoculation (Fig. 7a, c, d), which was statistically significant versus VLA1553-exposed animals on Day 1 post-exposure (*p* < 0.01). Furthermore, there was a trend towards a larger increase in monocytes in WT CHIKV- compared to VLA1553-exposed animals (Fig. 7b) at day 6 and day 7, but for these there was no statistically significant difference compared to baseline (day 1) in either group (WT group too small and large variation in the VLA1553 group).

**Fig. 8:**
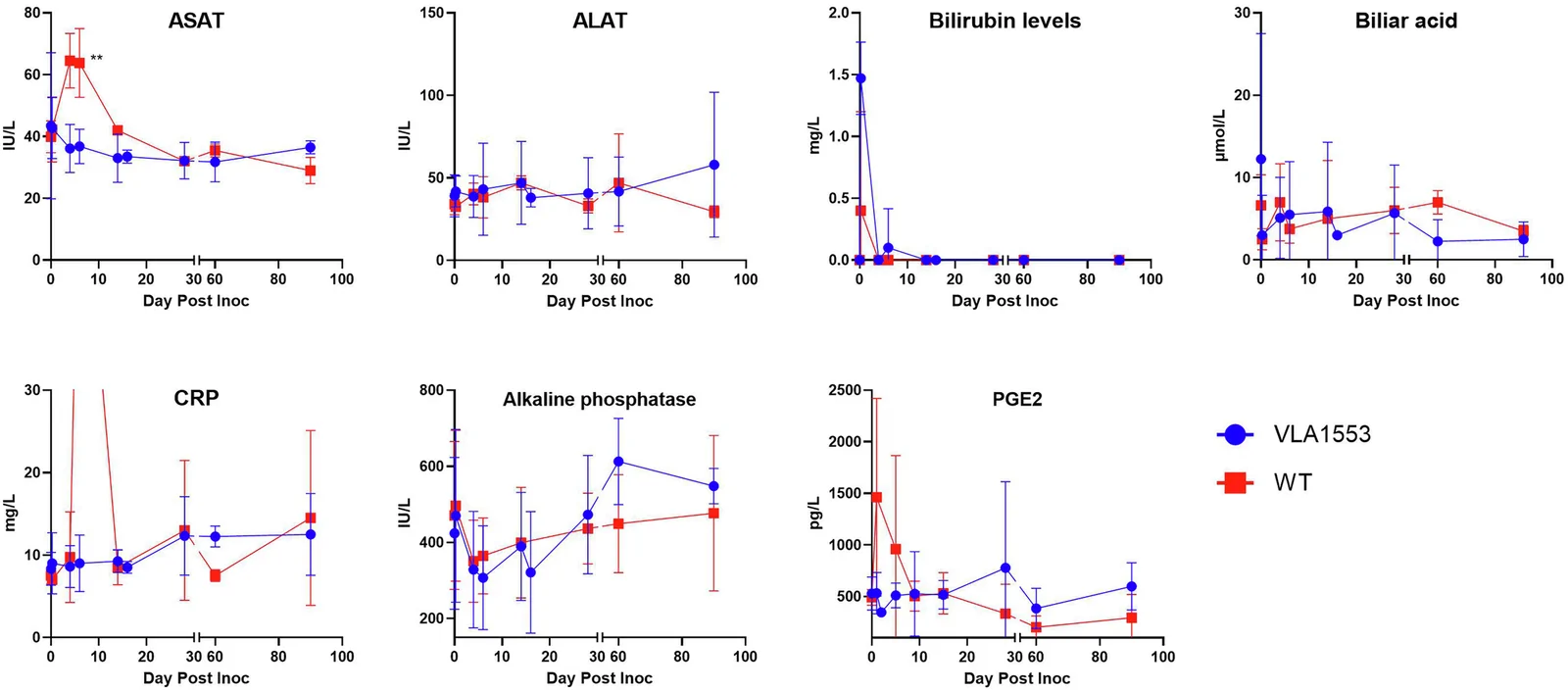
Blood biochemistry of VLA1553- and CHIKV-exposed NHPs. Baseline values were obtained from the mean of 2 to 3 blood samples obtained before the exposure of the animals to CHIKV. Statistics are based on peak titers in each animal (irrespective of time point) of WT versus VLA1553 using the Mann–Whitney test indicated by asterisks (*p < 0.05, **p < 0.01, ***p < 0.001).

Liver enzyme levels remained largely unchanged following VLA1553 exposure, with no significant change post-inoculation in ASAT or ALAT across animals. However, there was 1 animal with elevated ALAT levels; with already an app. 1.5-2-fold higher baseline level than all other animals, which showed a 2-fold increase in ALAT on Day 6 and Day 14 compared to baseline and 2–3-fold increase compared to NHPs exposed to WT CHIKV, but levels returned close to baseline by Day 90 (Fig. 8). In contrast, WT CHIKV exposure resulted in transient increases in liver enzymes, with ASAT elevated in all four animals on Day 4 and Day 6 (approximately 1.5- to 1.8-fold higher than baseline) and ALAT increased in one animal early after exposure (approximately 1.6- to 1.9-fold higher than baseline). The increases in WT CHIKV-exposed animals were transient and generally returned to baseline values by Day 14 (although in 2 of 4 WT CHIKV-exposed animals, no measurements were taken after Day 6).

Markers of hepatic function and inflammation remained largely stable in both groups. VLA1553 and WT CHIKV exposure did not affect total bilirubin levels, which remained below the detection limit for most animals in each group, and all recorded values remained very low. Similarly, there were no marked effects upon VLA1553 or WT CHIKV exposure on bile acid or alkaline phosphatase levels. C-reactive protein (CRP) levels were generally comparable between groups, aside from a single WT CHIKV-exposed animal showing a pronounced transient increase on Day 6 that was 5- to 10-fold higher than in the other animals in that group. Plasma prostaglandin E_2_ (PGE_2_) levels remained stable in VLA1553-exposed animals, with only one isolated elevation observed in one animal with PGE_2_ 2- and 3-fold higher than the mean values for the other animals on Day 9 and Day 28, respectively. In contrast, most WT CHIKV-exposed animals exhibited marked increases in PGE_2_ early after exposure, with 3 of 4 animals showing 4.5- to 5-fold increases from baseline in PGE_2_ levels between Day 1 and Day 5. Overall, VLA1553 showed minimal impact on inflammatory and hepatic biomarkers (Fig. 8).

### Lower and transient proinflammatory cytokine profile with VLA1553 in comparison to WT CHIKV

**Fig. 9:**
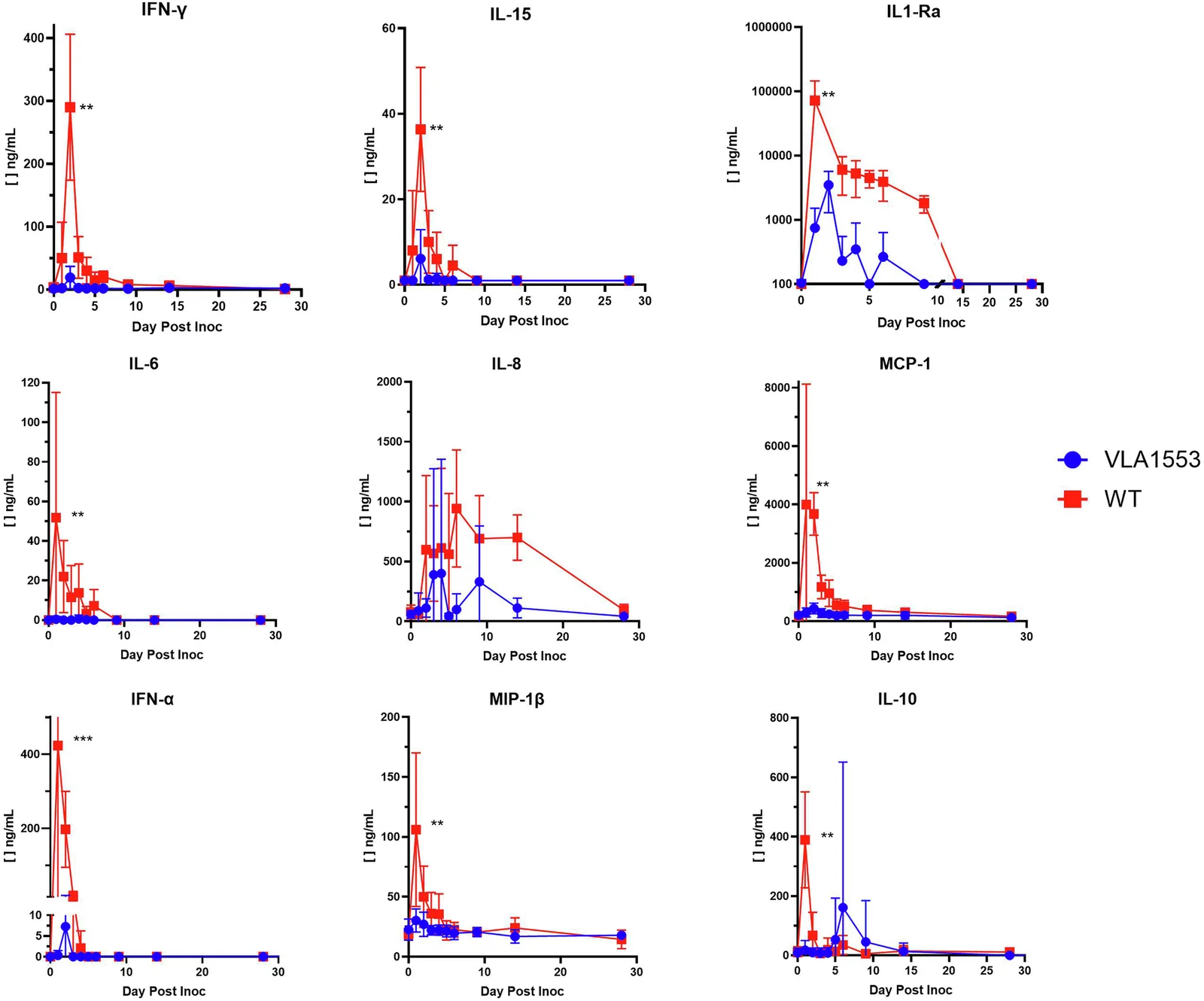
Cytokine expression profile in WT CHIKV- and VLA1553-exposed NHPs. IFN-γ, IL-10, IL-15, IL-6, and IL-8 were assessed by Milliplex assay. IL-1Ra, MCP-1, IFN-α, and MIP-1ß were assessed using the Cartaplex assay. Cytokine expression levels were determined until day 60 post-exposure. Levels are only shown until day 28 post-exposure, as baseline levels were reached by this time point. Mean of cytokine measurements from animals exposed to VLA1553 and WT CHIKV in blue and red, respectively. To note, the number of animals in the group exposed to VLA1553 was sufficient to assess potential variations in cytokine/chemokine levels, but the low number of control animals receiving WT CHIKV limited the statistical power of the comparison. Statistics are based on peak titers in each animal (irrespective of time point) of WT versus VLA1553 using the Mann–Whitney test indicated by asterisks (*p < 0.05, **p < 0.0, ***p < 0.001).

In CHIKV infection of both humans and macaques, viral load and severity of disease are strongly correlated to the upregulation of a number of cytokines, including IFNs, IL-6, IL-1Ra, TNF-α, and MCP-1^21,31,32^. In general, peak cytokine expression coincided with peak viremia at day 2 post-exposure. VLA1553 exposure was associated with a markedly reduced and short-lived cytokine response compared to WT CHIKV exposure (Fig. 9). Peak cytokine expression coincided with peak viremia at Day 2 in both groups; however, VLA1553 exposed animals exhibited significantly lower peak levels and a transient response limited to Day 2 for key cytokines, including IFN γ, IL 15, IL-1Ra, MCP 1, IFN α, and MIP 1β. This is in agreement with the reduced clinical symptoms and reduced plasma viremia that we have described in VLA1553-exposed NHPs compared to those exposed to WT CHIKV. In contrast, WT CHIKV induced higher and more sustained expression, beginning at Day 1, peaking at Day 2, and persisting for 3–5 days.

IL-8 responses occurred later in both VLA1553- and WT CHIKV-exposed animals, but remained lower in magnitude and shorter in duration following VLA1553 exposure. One VLA1553-treated animal showed on days 3 and 4 post-exposure 2 to 3 times higher IL-8 levels than WT CHIKV-exposed animals, which returned to baseline on Day 5. IL-6 was largely undetectable in VLA1553-exposed animals, whereas WT CHIKV induced robust expression lasting up to Day 6 in 3 of 4 animals. Similarly, IL-10 levels remained low following VLA1553 exposure but were markedly increased in WT CHIKV exposed animals at peak viremia, although 1 animal exposed to VLA1553 showed an increased level of IL-10 from Day 5 to 9 that peaked on Day 6 at 4-fold higher levels than observed for WT CHIKV-exposed animals at 1-day post-infection (Fig. 9). Overall, VLA1553 elicited a substantially attenuated and shorter cytokine response compared to WT CHIKV, consistent with reduced viremia and milder clinical presentation.

### Attenuated tissue pathology in VLA1553-exposed animals

Histological findings in the spleen, axillary lymph nodes (i.e., those draining the CHIKV inoculation site), left extensor digitalis communis, and liver are described in the following paragraphs.

In both groups, the spleen showed a moderate increase in activation over time (Supplementary Fig. 2), which was more intense in WT CHIKV-exposed animals (Day 90) than VLA1553-exposed animals, as highlighted by the relative cellularity, lymphocytes, follicles, and white pulp (index 2.5 [WT CHIKV-exposed] and 1.5 [VLA1553-exposed]) (Table 1). Animals exposed to VLA1553 returned to a steady state, whereas those exposed to WT CHIKV showed signs of persistent activation.

**Table 1:** Analysis of tissue pathology by hematoxylin and eosin staining

		VLA1553						WT CHIKV	
Organ		Day 2	Day 6	Day 14	Day 28	Day 60	Day 90	Day 6	Day 90
Spleen	Cellularity, lymphocyte, follicle, white pulp	0.5	1	2	1	2	1.5	1	2.5
	Cellularity, increased, macrophages, red pulp	1.5	0	0.5	1	0.5	0.5	1	0.5
Axillary lymph node	Length	4352	5322	6027	4389	3649	5149	2783	5631
	Width	2891	3261	4862	1916	1787	2691	1954	3684
	Tingible body macrophages, germinal center	1.5	0.5	1.5	2	1	0.5	0	2
	Follicular hyperplasia	1	0.5	2.5	2	1	1.5	1	2.5
Left extensor Digitalis communis	Macrophage infiltration	0.5	0	0	0	0	0	0	0
	Basophilic fibers	0.5	0	0	0	0	0	0	0
Liver	Microvesicular and macrovesicular, hepatocytic inclusion, centrolobular	2	2	2	1.5	0.5	1.5	0.5	1
	Lymphocytic foci, multifocal	0.5	0	0	0	0.5	1.5	0.5	0.5
	Hepatocyte dissociation	0.5	0	0	0	0	0.5	0	1
	Centrolobular congestion	0.5	1.5	1	1	0	0.5	1	1

Two slides per time point and tissue were analyzed and expressed as pathology score derived from the International Harmonization of Nomenclature and Diagnostic Criteria system^42^ (0 = no lesion, 1 = mild, 2 = moderate, 3 = marked, 4 = severe).

Of the animals exposed to VLA1553, 2 animals were sacrificed at each timepoint, and of the animals exposed to WT CHIKV, 2 animals were sacrificed at Day 6 and Day 90

Mean of values of each time point and organ are shown.

VLA1553 = 3.2 × 10^6^ TCID_50_ VLA1553 (intramuscular)

WT CHIKV = 3.2 × 10^6^ TCID_50_ WT CHIKV LR2006-OPY1 (intramuscular)

The axillary lymph nodes showed an increase in size (both length and width) over time (Table 1). In NHPs exposed to VLA1553, a moderate increase on Day 14 to Day 28 had returned to a steady state at Day 90, whereas in those exposed to WT CHIKV, there was an increase in activity (as indicated by the number of macrophages) on Day 90 compared to Day 6 (Supplementary Fig. 3).

The left extensor digitalis communis showed very limited pathological signs (Supplementary Figure 4) in either group. Minimal lesions characterized by minimal macrophage infiltration and basophil fibers were observed on Day 2 in VLA1553-exposed animals, but no samples were available from animals exposed to WT CHIKV for comparison at this time point (Table 1).

In the liver, minimal to moderate microvesicular and macrovesicular hepatocytic centrolobular to lobular inclusions (steatosis) were observed, especially in VLA1553-exposed animals on Day 2 (Table 1). Minimal foci of lymphocytes were observed in each group, with a small increase at the late stage in animals exposed to VLA1553. Observed steatosis was minimal to mild and could be related to the physiological state of the animals. One animal exposed to VLA1553 presented some dissociation at Day 90 that coincided with an increase in ALAT, and hepatocyte dissociation was observed especially on Day 90 in WT CHIKV-exposed animals (Table 1 and Supplementary Fig. 5). No hemosiderosis was observed in either group.

## Discussion

The aim of this study was to assess the risk for viral persistence after vaccination with the live-attenuated vaccine virus VLA1553 in comparison to WT CHIKV over a period of 90 days, through the administration of a high dose of VLA1553 or WT CHIKV to NHPs. The *Macaca fascicularis* species was used to model the human situation due to its susceptibility to CHIKV infection and its capacity to reproduce the viral, clinical, and pathological features observed in humans, which has been demonstrated previously^21^. In contrast to the study published by Labadie et al.^21^, where NHPs were inoculated via the i.v. and i.d. route at a dose of 10^3^ PFU this study, NHPs were inoculated i.m., the route of administration of IXCHIQ^®^. Furthermore, to mimic a “worst-case” scenario, the dose level we used (3.2 × 10^6^ TCID_50_) was 1 log higher than the highest human dose of VLA1553 used in Phase 1 clinical development^14^ and 2 logs higher than the dose used in Phase 3 clinical development^24,25,33^ (the actual dose of licensed VLA1553 is not less than 10^3^ TCID_50_, per the IXCHIQ package insert (https://www.ema.europa.eu/en/documents/referral/ixchiq-article-20-procedure-product-information_en.pdf)). Regarding clinical observations, fever was only observed transiently following exposure of NHPs to VLA1553 and coincided with peak viremia on Day 1 and 2 while WT CHIKV exposure resulted in fever from Day 1 to Day 6 post-exposure. These findings agree with previously published data, despite different administration routes and doses^21,22^. Also in agreement with the previous studies, WT CHIKV viremia peaked at approximately 10^9^ RNA copies/mL. RNA titers in NHPs receiving VLA1553 were approximately 3 logs lower during peak viremia on Days 2 and 3 post-exposure than in animals receiving WT CHIKV (*p* < 0.01). Interestingly, in this study, VLA1553 peak viremia was not delayed by 1 or 2 days as observed previously^22^. The delayed viremia of VLA1553 observed in Roques et al.^22^ in comparison to our study may be due to differences in vaccine virus production (BHK-21 vs. Vero cells) or virus quantification methods (plaque assay vs. TCID₅₀) and thus the administered doses. A clear relationship between the inoculation dose and the period and magnitude of viremia in NHPs was demonstrated previously for WT CHIKV^21^.

In clinical studies, VLA1553 vaccination was considered to be reactogenic but safe, with a favorable risk-benefit evaluation that will vary depending on factors such as chikungunya virus transmission intensity^34^, whereas WT CHIKV infection can lead to the acute and chronic stages of chikungunya disease described earlier. The high dose of VLA1553 that we used was also hypothesized to increase the chance of detecting viral RNA in the tissues, as it was demonstrated that the initial CHIKV inoculation dose can be a key driver of disease progression and tissue persistence^21^.

In humans, CHIKV infection can result in viral loads ranging from 10^3^ to 10^10^ RNA copies/mL^26–28^. It is noted that the viral loads measured may depend on the time point relative to infection and onset of symptoms. Plasma viremia after vaccination with VLA1553 in a Phase 1 clinical trial peaked at Day 3, with the highest mean GCE (or RNA copies) occurring in the highest dose group (receiving 3.2 × 10^5^ TCID_50_), determined at 2.3 × 10^5^ GCE/mL. The GCEs in low (receiving 3.2 × 10^3^ TCID_50_) and medium (receiving 3.2 × 10^4^ TCID_50_) dose groups were considerably lower, reaching mean titers of 7.4 × 10^4^ and 8.9 × 10^4^ GCE/mL, respectively^14^. The highest measured viremia was 1.88 × 10^6^ GCE/mL.

Tissue analyses showed limited and more sporadic presence of VLA1553, except for the immune organs (spleen and lymph nodes), with VLA1553 RNA detected in the spleen from Day 2 to Day 60 post-exposure. However, RNA levels were approximately 2 log lower than in WT CHIKV-exposed animals, and replicating virus was only detected on Day 2 in the spleen but in no other tissue, in contrast to WT CHIKV-exposed NHPs, in which replicating virus was also detected in the axillary lymph node and the digit abductor. Our data are in agreement with Labadie et al.^21^, who observed WT CHIKV persisting in the lymphoid tissues for up to 3 months and also recovered replicating WT CHIKV at day 44 post-exposure. We do not have data for this time point, but in our study, no replicating WT CHIKV was observed 3 months post-exposure. A limitation of this study is that CHIKV localization in the cytoplasm of mononuclear cells in the spleen and lymph nodes was not evaluated, although macrophages were shown to play an important role in viral persistence in macaques^21^. Importantly, in the brain, no VLA1553 RNA was detected, while WT CHIKV RNA was detected in the choroid plexus, which was also shown previously^35^. The level of virus detected in the tissues was consistently higher in WT CHIKV-exposed animals than in those receiving VLA1553. When it comes to viral shedding, in this study, no CHIKV RNA was detected in body fluids (synovial and cerebrospinal fluid or urine) of NHPs exposed to either WT CHIKV or VLA1553, contrary to the study from Labadie et al.^21^, where WT CHIKV was detected in cerebrospinal fluid until 55 days post-infection. Differences between this study and Labadie et al.^21^ may be attributed to the different inoculation routes, i.m. versus i.v. administration, respectively. WT CHIKV was detected in saliva and vaginal fluid, while VLA1553 was detected in a low number of animals, which also contributed to blood contamination and menstruation, respectively. As samples were not tested for replicating CHIKV, we cannot draw conclusions on the actual viral shedding of live, replicating CHIKV.

In terms of hematology and blood biochemistry parameters, the main differences between groups were lymphocytopenia, increased liver enzymes, and increased PGE_2_ in WT CHIKV-exposed NHPs. Only limited information on hematopoietic cell counts is available in humans, but leukopenia is often observed during CHIKV infection^36^. Additionally, in WT CHIKV-exposed animals, CRP, which is considered to be a robust marker for inflammation, was elevated in 1 animal, although it has previously been shown that even if statistically increased in WT CHIKV-exposed cynomolgus macaques, this is not a very sensitive marker for CHIKV infections^21^. PGE_2_ values are related to age (in our study, the animals were comparable in age to those described by Ebersole et al.^37^, i.e., young to adolescent) and reflect the immune status of the liver. The increase in PGE_2_ in NHPs exposed to WT CHIKV may be related to the high level of viral replication observed in these animals and could be suggestive of an enhancement of the pro-inflammatory response to viral-induced arthritis^38^. In VLA1553-exposed animals, only a single PGE_2_ value at a single timepoint was raised, with all other values in this group being within the normal range as described in humans or in rhesus macaques (500 to 1000 pg/mL)^37^. Thus, no signs of immune activation related to arthritis were observed in animals inoculated with VLA1553.

In both humans and macaques, CHIKV load and severity of disease are strongly correlated to the upregulation of cytokines^21,31,32^. It has previously been shown that 7 cytokines are particularly upregulated in NHPs after WT CHIKV infection^22^, although in the earlier study, NHPs received WT CHIKV and vaccine virus via the s.c. route, compared to our study in which WT CHIKV and VLA1553 vaccine virus were administered via the i.m. route and at an approximately 2-fold higher dose level. We analyzed a large set of analytes to assess the profiles of cytokines, chemokines, and growth factors in both groups of animals. Cytokine expression was observed for IFN-γ, IL-6, IL-8, IL-10, IL-15, IL-1Ra, MCP-1, IFN-α, and MIP-1β in both groups, with peak expression generally coinciding with peak viremia or soon after. Expression of these cytokines was higher both in peak magnitude and duration in WT CHIKV-exposed animals than in those receiving VLA1553, which reached significance during peak viremia. In our study, IL-6 expression was highest during peak viremia and decreased to undetectable levels by day 9, which was not consistent with the findings of Labadie et al.^21^, who showed that high IL-6 levels persisted until Day 6 post-infection. In our study, MCP-1, which is involved in monocyte/macrophage recruitment, also peaked on Days 2 and 3 post-WT CHIKV infection and declined thereafter, while Labadie et al.^21^ detected high levels of MCP-1 for 15 days post-infection. This could also be a consequence of a different route or dose of inoculation used by Labadie et al.^21^. Overall, the expression of pro-inflammatory cytokines and chemokines usually associated with WT CHIKV disease was much lower and more limited in duration in VLA1553-exposed compared to WT CHIKV-exposed animals. Furthermore, there was no detection of any significant quantities of pro-inflammatory cytokines TNF-α or IL-17a in any animals in either group. This is consistent with the lack of arthritis symptoms in the NHPs, with IL-17a expression identified as one of the key contributors to arthritic inflammation in rheumatoid arthritis^39^.

In terms of histology, inoculation of animals with VLA1553 or WT CHIKV had little effect on the spleen, axillary lymph node (the draining lymph node of the inoculation site), liver, and extensor digitalis communis muscle (the site of inoculation). There was minimal to moderate lymphoid activation in WT CHIKV-exposed NHPs, which was less apparent in VLA1553-exposed animals. Observations of hepatocyte dissociation could be related to the infection, and the lack of hemosiderosis in our study contrasts with previous work following intravenous inoculation^21^; which may again be explained by the different route of inoculation. Furthermore, in the previous study, no histological change or activation before Day 6 to Day 7 was observed in WT CHIKV-exposed animals, whereas we observed faint signs of liver steatosis at Day 2 in VLA1553-exposed animals. However, this is likely to be related to the body fat status of the animals in VLA1553-exposed animals and a slight increase of lymphoid macrophages in 1 animal.

Limitations of this study include the imbalanced sample size, with relatively few WT CHIKV-exposed NHPs. Further, the limited number of animals and sampling time points in the WT CHIKV group did not allow for a complete comparison of the dynamics of the WT CHIKV and VLA1553.

In conclusion, despite the dispersion of the virus through the blood circulation and detection of very low levels of vaccine virus at early time points after VLA1553 administration, no significant viral persistence was observed beyond Day 28, except for low levels of viral RNA in the immune organs that drive the immune response. Replicating VLA1553 was only detected in the spleen on Day 2 post-inoculation, with none shown in the brain, joints, or other tissues. The study confirmed the attenuation of the vaccine strain, and there were no critical observations regarding the live-attenuated VLA1553 vaccine in an NHP model.

## Methods

### Animals and groups

Sixteen naïve young adult female cynomolgus macaques (*Macaca fascicularis*), specifically dedicated to this study, were imported from Mauritius to the Atomic Energy Commission (CEA) facilities in Fontenay-aux-Roses, France. The NHPs were a homogeneous group in terms of age and weight, and prior to inoculation on Day 0, NHPs in each group were comparable in terms of age (2.7 to 4.4 years in VLA1553-exposed animals and 2.6 to 4.5 years in WT CHIKV-exposed animals) and body weight (2.9 to 4.7 kg, respectively). The individual animals were characterized for major histocompatibility complex (MHC) haplotype^40^. Sex was not considered a biological variable, and the findings from the female NHPs included in the study were considered to be applicable to male animals also.

### Animal housing

Animals were housed and handled according to Directive 2010/63/CE and French law “décret 2013-118” from 1 February 2013. They were housed in social groups except during the first 2 weeks after virus inoculation, when they were housed individually in adjoining cages, allowing social, visual, and auditory interactions. Cages were in a climate-controlled room (temperature 21 ± 1°C and relative humidity 55 ± 5%) with a 12-hours light/dark cycle. The head of the veterinary staff was empowered to interrupt the protocol in the event of animal pathology or suffering. Animals were fed daily *ad libitum* with specific food and fruits, and enrichments (toys and special “hidden” foods) were provided.

### Infection of NHPs with WT CHIKV and VLA1553

Animals were exposed to VLA1553 or WT CHIKV on Day 0. Sedated animals were placed in a dorsal recumbency with their muscles relaxed. Immediately before administration of the virus dose, the zone of skin at the intramuscular injection site in the left arm at 7–8 cm from the wrist was shaved and cleaned with 70% ethanol. Each animal received one injection of 0.1 mL of VLA1553 (3.2 × 10^6^ TCID_50_) (VLA1553-exposed animals) or WT CHIKV LR2006-OPY1 (3.2 × 10^6^ TCID_50_) (WT CHIKV-exposed animals) intramuscularly in the arm. Twenty-four hours post-exposure, each macaque was physically examined for fever, erythema, edema, and erosion.

### Anesthesia of NHPs for immunization and sampling procedures

Immunization and sampling procedures were performed by CEA staff under the supervision of the veterinary staff. For all procedures, animals were sedated with Tiletamine/Zolazepam (Zoletil, Virbac, 06517 Carros, France, 5 mg/kg [0.05 mL/kg], intramuscular route) in accordance with institutional guidelines. The rationale for using Zoletil and not Ketamine was to avoid stress of the animals, as the injection volume needed for Zoletil was very small and anesthesia was fast with the animals’muscles being fully relaxed. For euthanasia, after sedation, animals were humanely euthanized by intravenous injection of pentobarbital (180 mg/kg).

### Measurements of body temperature

Body temperature was monitored using STAR ODDI chips (www.star-oddi.com) implanted subcutaneously in the upper back, between the scapulae. Temperature was recorded every 2 hours, and the chips were removed at euthanasia to collect the data.

### CHIKV quantification in plasma and other body fluids

Total CHIKV RNA in NHP plasma, saliva, vaginal fluid, cerebrospinal fluid, synovial fluid, and urine was quantified using a CHIKV-specific RT-qPCR. CHIKV titers in plasma were assessed as early as 6 oursh post-exposure over a period of 9 days for WT CHIKV-exposed and VLA1553- exposed animals. Saliva of animals infected with CHIKV was analyzed on Days 2, 4, 6, 60, and 90 for WT CHIKV-exposed animals and on Days 2, 4, 6, 9, 13, 30, 60, and 90 for VLA1553-exposed animals. Vaginal fluid was analyzed on Days 2, 4, 6, 60, and 90 for WT CHIKV-exposed animals and on Days 2, 4, 6, 30, 60, and 90 for VLA1553-exposed animals. Urine, synovial, and cerebrospinal fluid were collected and analyzed at euthanasia time points. The same assays were used for the detection of both WT CHIKV RNA and VLA1553 RNA. Two RT-qPCR methods were used, classical and ultrasensitive RNA extraction, essentially as described previously by Roques et al.^23^. These two methods differed in the RNA extraction procedure to allow RNA extraction from standard samples with high viral loads (classical RNA extraction) and from samples with expected low viral loads (ultrasensitive RNA extraction). Classical RNA extraction and RT-qPCR were used for plasma samples with an expected high viral load between Day 0 and Day 4 post-infection. The Lower Limit of Quantification (LLOQ) was 5000 copies/mL (62.5 copies/reaction), and the sensitivity of the assay (Limit of Detection, LOD_95_) was 720 copies/mL (9 copies/reaction), which was thus also the Lower Limit of Detection (LLOD). Ultrasensitive RNA extraction and RT-qPCR allowed better sensitivity in body fluids with a low viral load, including plasma from Day 5 to Day 10 post-infection and saliva, urine, vaginal, and cerebrospinal fluids. The LLOQ for plasma samples was 500 copies/mL (42 copies/reaction), and the sensitivity of the assay (LOD_95_) was 60 copies/mL (5 copies/reaction), which was thus also the LLOD. Detection limits for the other body fluids were assumed to be in a comparable range but were not separately determined due to the very limited amount of material available.

### Necroscopy and CHIKV quantification in tissue samples

Tissue lysates were generated by mechanical disruption (Precellys system) of tissue samples using lysis buffer (RA1 Machery–Nagel, ref# 740961.500) + 4% TCEP (Machery–Nagel, ref# 740395.107), including brain and lymphoid tissues and a small part of the muscle tissues (Biceps Brachi). For all remaining tissues, an improved lysis protocol was used with NucleoZOL buffer (Machery–Nagel, ref# 740404.200). Thus, the hand and finger muscle tissue and skin, as well as all replicates for other tissues, were performed using NucleoZOL. All lysed tissues were further processed with a Precellys system, using tubes with ceramic beads for soft tissues (Bertin Technologies, ref# 03961-1-0092) and tubes with metal beads for hard tissues (Bertin Technologies, ref# 03961-1-0012). The tissue lysates were diluted to 100 mg of tissue/mL in Macherey–Nagel lysis buffer, aliquoted, and stored at −80 °C until extraction.

### CHIKV RNA quantification in tissues

RT-qPCR was performed as described previously^21^, where CHIKV RNA RT-qPCR quantification cycle (Cq) measurements were compared to cellular GAPDH (endogenous control) RNA. All reactions were 90–105% efficient, thus, each CHIKV sample was normalized using a GAPDH sample and the relative copy number for each sample was calculated with the following equation: Relative copy number = 2-(Cq CHIKV–Cq GAPDH) RNA levels are expressed as the ratio of CHIKV RNA to cellular GAPDH RNA in the following tissues: spleen, liver, lymph nodes, left arm muscle (site of injection), left hand/finger muscle, brain (frontal encephalon, mesencephalon, cerebellum, choroïd plexus), joints (left knee and elbow capsula), skin at the inoculation site and on the contra-lateral arm, cervix, vagina. The limit of detection for CHIKV/GAPDH RNA ratio is <1E-06 for all tissues except for the brain, with a detection limit of 5 × 10^−6^, and liver as well as peripheral lymph nodes with 3 × 10^−6^, respectively.

### Detection of replicating CHIKV in tissues

A Vero cell-based TCID_50_ assay with a pre-amplification step on mosquito cells was used to quantify very low amounts of CHIKV in selected tissues. Although this method is more sensitive than a conventional TCID_50_ assay where no pre-amplification on mosquito cells is performed, this method is not quantitative due to the pre-amplification step. Thus, results are only reported as the presence or absence of replicating CHIKV. Vero E6 cells and C6/36 cells were cultured in DMEM + 10% fetal calf serum + 1% sodium pyruvate. Amplification of WT CHIKV and VLA1553 from respective NHP tissue lysates on C6/36 cells was performed using undiluted tissue samples as well as 1:10, 1:100, and 1:1,000 dilutions, and were tested in triplicate. Amplification on C6/36 cells was allowed for 3 days at 28 °C/5% CO_2_. After 3 days, each well was checked under the microscope, and from each triplicate of the tissue sample dilutions (undiluted, 1:10, 1:100, and 1:1,000), supernatants were pooled for titration on Vero E6 cells. The pooled supernatants from amplification on C6/36 cells were then analyzed in a conventional Vero cell-based TCID_50_ assay. Two hours after seeding of 2.5 × 10^5^ Vero E6 cells per well, the 5-fold serial dilutions prepared from the C6/36 supernatant pools ranging from 1:5 to 1:1,220,703,125 were added onto the Vero E6 cells. The 96-well plates were incubated for 72 h at 37 °C/5% CO_2_. Viral replication was assessed by observation of cytopathic effect under the microscope, and in addition, cell viability was assessed using CellTiter 96® Aqueous Non-Radioactive Cell Proliferation Assay (MTS) (Promega) following the manufacturer’s instructions.

### Hematology and biochemistry analyses

Hematology analyses (complete blood counts) were performed using a DxH800 (Beckman Coulter). Serum alanine aminotransferase (ALAT), aspartate aminotransferase (ASAT), alkaline phosphatase, bilirubin, and bile acids were analyzed with standard kits (Siemens) and CRP with a canine kit (Randox), using an ADVIA1800 chemistry system (Siemens). PGE_2_ was analyzed using a prostaglandin E2 ELISA kit-Monoclonal (Cayman Chemical, Ann Arbor, MI, USA)^41^

### Cytokine and chemokine profiles of inflammation and innate immune responses

Plasma was assessed for cytokines and chemokines using two different multiplex systems, the 37-Plex NHP ProcartaPlex™ Panel and the Milliplex Map NHP Cytokine Magnetic Bead Panel, and analyzed by a Bioplex 200 system. The following analytes were tested: brain-derived neutrotrophic factor, B lymphocyte chemoattractant (CXCL13), Eotaxin (CCL11), fibroblast growth factor-2 (FGF-2), granulocyte colony-stimulating factor (G-CSF) (CSF-3), granulocyte macrophage colony-stimulating factor GM-CSF, Granzyme B, interferon- α (IFN-α), IFN-γ, interleukin-1β (IL-1β), IL-10, IL-12, IL-13, IL-15, IL-17, IL-18, IL-1Ra, IL-2, IL-23, IL-4, IL-5, IL-6, IL-7, IL-8 (CXCL8), IP-10 (CXCL10), interferon-inducible T cell alpha chemoattractant (I-TAC) (CXCL11), monocyte chemoattractant protein-1 MCP-1 (CCL2), membrane immunoglobulin (CXCL9), macrophage inflammatory protein (MIP-1α) (CCL3), MIP-1β (CCL4), nerve growth factor (NGF-β), platelet-derived growth factor (PDGF-BB), soluble cluster of differentiation-ligand (sCD40L), stem cell factor, stromal cell-derived factor-1α (SDF-1α) (CXCL12a), tumor necrosis factor-α (TNF-α), vascular endothelial growth factor-A (VEGF-A), VEGF-D, and transforming growth factor (TGF-α).

### Histology

The histological examination included the spleen, liver, axillary lymph node, and left extensor digitalis communis muscle. Organs were fixed using 10% paraformaldehyde buffered solution for 24 hours, then embedded in paraffin (FFPE), sliced into 5 μm slides, and stained with hematoxylin and eosin (H&E), and kept at room temperature. Slides were digitalized at ×20 with 3DHistech Pannoramic SCAN II (Sysmex, Roissy, France), and all histopathology examinations were performed by a board-certified veterinary pathologist (QP, DVM, DESV-AP). The grading system was adapted from the InHand (International Harmonization of Nomenclature and Diagnostic Criteria) pathology system for mice and rats^42–44^. Each criterion was attributed with an intensity moderator (0 = no lesion, mild = 1, moderate = 2, marked = 3, severe = 4).

### Statistics

Statistical analysis was performed under the responsibility of Valneva using the GraphPad Prism 6 software (GraphPad Software). A Kruskal–Wallis test was used for multiple comparisons, followed by a 2-tailed Mann–Whitney U test to analyze differences between groups for immune response kinetics (AUC). Significant differences were obtained for *p* < 0.05 (*), *p* < 0.01 (**), and *p* < 0.001 (***). All tests were performed with a 95% CI. Bonferroni’s correction was used for multiple comparisons.

### Study approval

The study was approved by the regional animal care and use committee (Comité Regional d’Ethique sur l’Expérimentation Animale Ile de France Sud, Fontenay-aux-Roses, France) (approval number A18_051, from 6 September 2018 and validated by the French MENESR under the number APAFIS#16755-2018091711094093v2 on 26 November 2018).

## Supplementary information

Roques et al., 2026_npjVaccines_VLA1553_supplements_REVISED_07JUL26.

## Data Availability

The datasets generated and/or analyzed during the current study are not publicly available due to commercial sensitivity but are available from the corresponding author upon request.
