# Supplementary material for: Limited tissue persistence confirms attenuation of live-attenuated chikungunya vaccine VLA1553 in non-human primates
Source: NPJ Vaccines. 2026 Jul 23;11:194. doi: 10.1038/s41541-026-01535-4 (PMC13687089; doi:10.1038/s41541-026-01535-4)
Supplement: Supplementary file 1 [file 41541_2026_1535_MOESM1_ESM.pdf]

SUPPLEMENTARY MATERIAL

Supplementary Table 1: CHIKV/GAPDH RNA ratio in the spleen and lymph nodes

					Lymph nodes											
	Spleen				Axillary				Inguinal				Nasopharyngeal			
	VLA1553		WT CHIKV		VLA1553		WT CHIKV		VLA1553		WT CHIKV		VLA1553		WT CHIKV	
Day	NHP #1	NHP #2	NHP #1	NHP #2	NHP #1	NHP #2	NHP #1	NHP #2	NHP #1	NHP #2	NHP #1	NHP #2	NHP #1	NHP #2	NHP #1	NHP #2
2	4.48E-03	5.08E-03[b]	ND	ND	8.73E-05	BLD	ND	ND	1.69E-05	BLD	ND	ND	BLD	BLD	ND	ND
6	4.90E-03[a]	3.79E-02[a]	5.00E-01[b]	1.40E+00[b]	8.80E-03	2.20E-04[a]	4.07E-01[a]	1.31E-01[b]	6.11E-04	4.82E-05	2.56E-01[c]	7.24E-02	BLD	1.13E-04	9.21E-03[a]	1.03E-01[a]
14	1.21E-01[a]	3.60E-03	ND	ND	1.14E-04	6.44E-05	ND	ND	8.83E-05	1.73E-04	ND	ND	3.22E-05	1.19E-05	ND	ND
28	6.60E-03	BLD	ND	ND	1.08E-03	BLD	ND	ND	BLD	2.41E-05	ND	ND	BLD	BLD	ND	ND
60	6.55E-04	1.20E-03	ND	ND	1.20E-02	1.09E-05	ND	ND	1.50E-05	1.44E-05	ND	ND	BLD	BLD	ND	ND
90	5.12E-04	8.17E-05	1.09E-02	2.48E-03	6.15E-03	BLD	4.26E-03[c]	4.13E-03	BLD	BLD	7.82E-03	1.08E-02	BLD	BLD	3.24E-03	4.62E-04

Of the animals exposed to VLA1553, 2 animals were sacrificed at each timepoint and in the animals exposed to WT CHIKV, 2 animals were sacrificed at Day 6 and Day 90
BLD, below limit of detection; ND, not done; NHP, non-human primate
VLA1553=3.2×10^6 $TCID_{50}$ VLA1553 (intramuscular)
WT CHIKV=3.2×10^6 $TCID_{50}$ WT CHIKV LR2006-OPY1 (intramuscular)
[a]No replicating WT CHIKV or VLA1553 were detected when assessed in the $TCID_{50}$ assay
[b]Replicating WT CHIKV or VLA1553 were detected in a $TCID_{50}$ assay
[c]Only 1 extraction was done because of very small tissue sample

Supplementary Figure 1 Hematologic parameters determined after WT CHIKV or VLA1553 exposure.

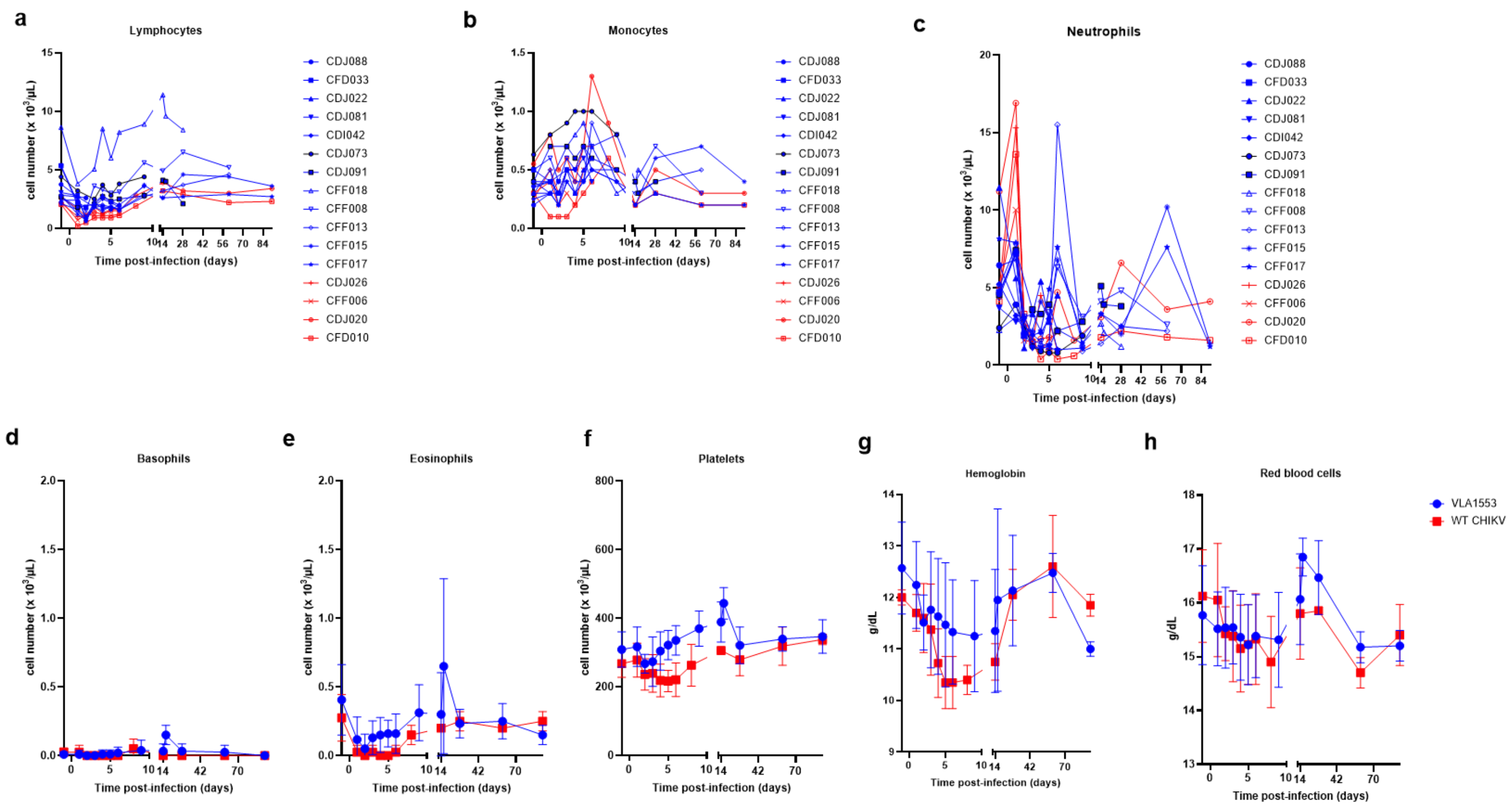

a – c Individual variations and numbers of **a.** lymphocytes, **b.** monocytes and **c.** neutrophils. **d – h** Mean values of cell numbers or concentrations (grams per deciliter) of **d.** basophils, **e.** eosinophils, **f.** platelets **g.** hemoglobin and **h.** red blood cells. Baseline values are shown as Day -1. Data are presented for VLA1553-exposed NHPs in blue and WT CHIKV-exposed NHPs in red.

Supplementary Figure 2 Histology of spleens in animals exposed to VLA1553 and WT CHIKV.

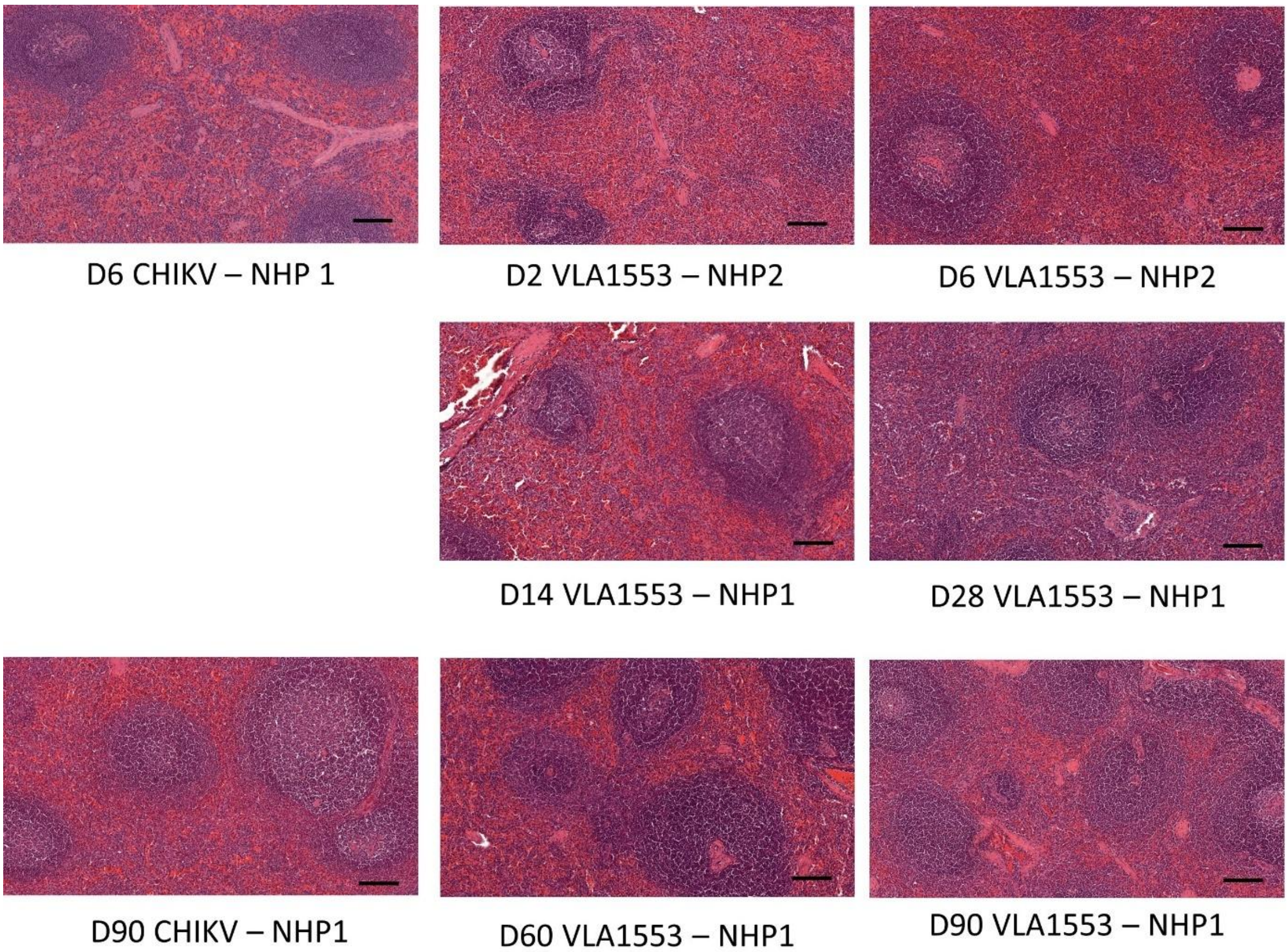

Spleens from VLA1553 exposed animals were examined on Days 2, 6, 14, 28, 60, and 90 and compared to spleens from WT CHIKV-exposed animals from Day 6 and Day 90. On day 90 WT exposed NHP1 shows moderate follicular hyperplasia. However, on day 28 VLA1553 exposed NHP1 shows minimal follicular lymphoid hyperplasia.

Supplementary Figure 3 Histology of axillary lymph nodes in animals exposed to VLA1553 and WT CHIKV.

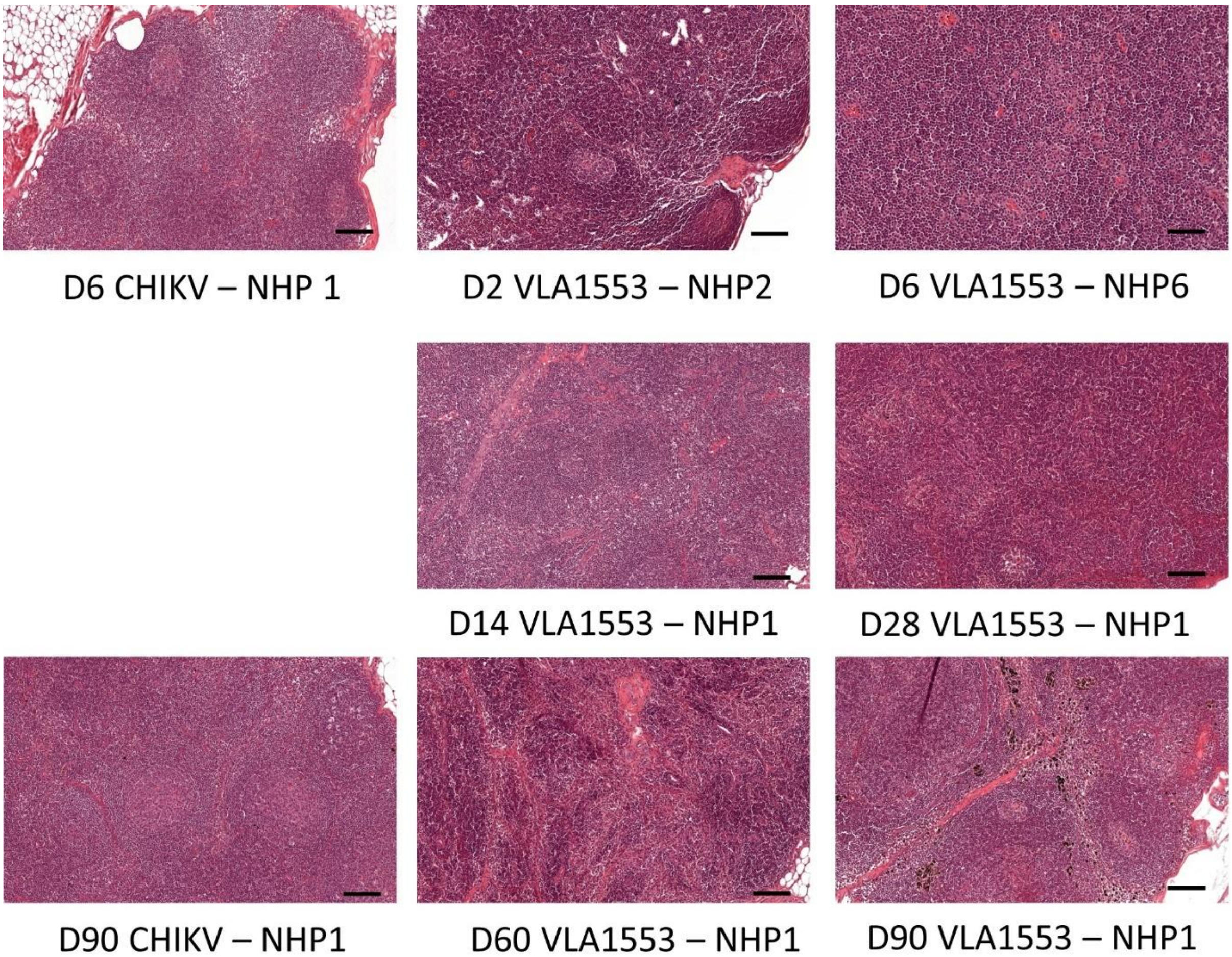

At day 90 WT CHIKV-exposed animal NHP1 shows enlarged germinal centers with follicular hyperplasia with few tangible bodies. VLA1553-exposed animal shows minimal follicular enlargement with minimal lymphoid hyperplasia at the same time point.

Supplementary Figure 4 Histology of extensor communis digitalis in animals exposed to VLA1553 and WT CHIKV.

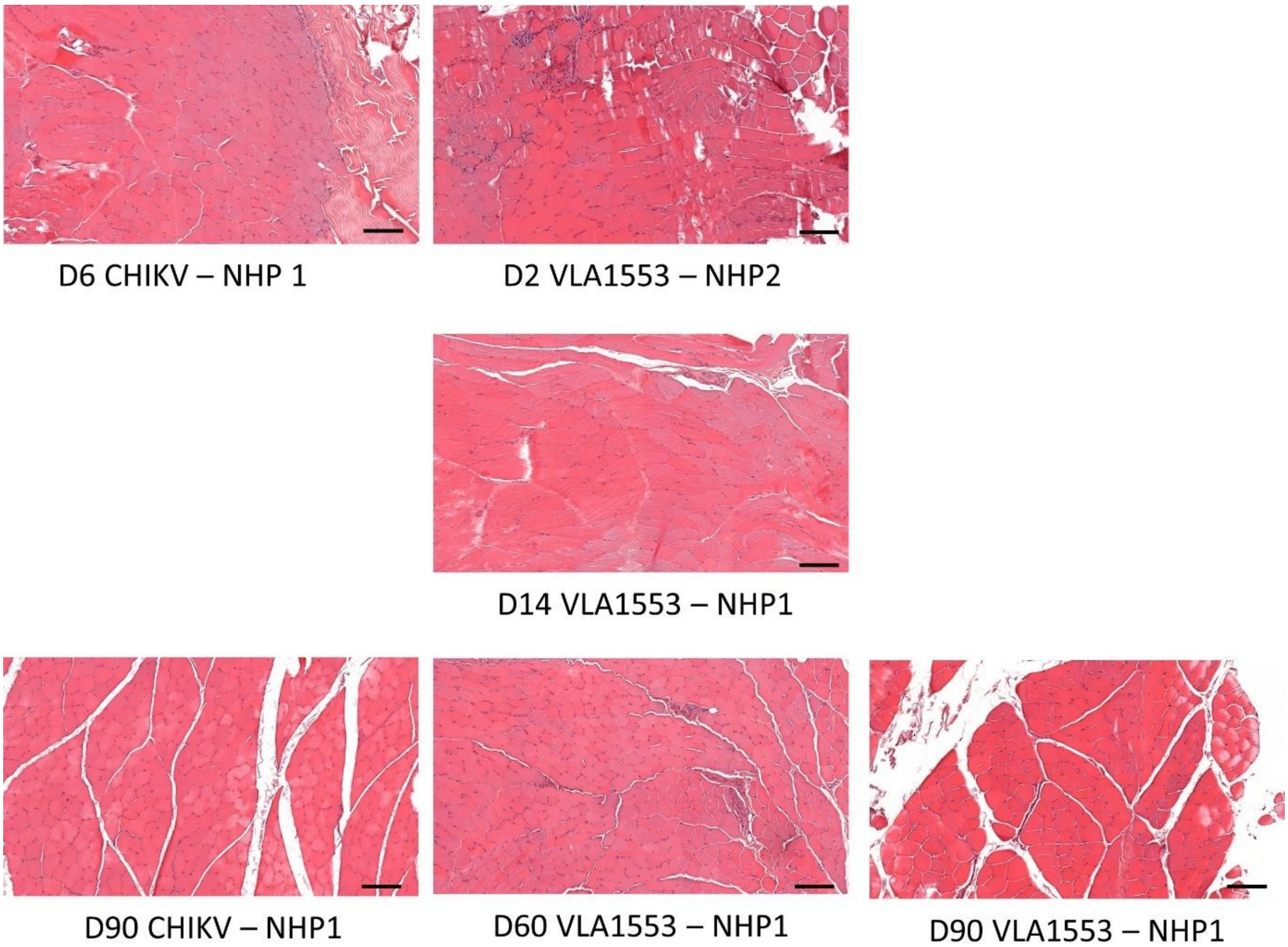

Extensor communis digitalis muscles from VLA1553 exposed animals were examined on Days 2, 6, 14, 28, 60, and 90 and compared to muscles from WT CHIKV exposed animals from Day 6 and Day 90. Due to scanning quality for VLA1553-exposed animals, muscles from Day 6 and Day 28 were examined under the microscope, thus scans are not shown. Minimal degenerative changes are observed in the muscle fibers of vaccinated animals.

Supplementary Figure 5 Histology of liver in animals exposed to VLA1553 and WT CHIKV

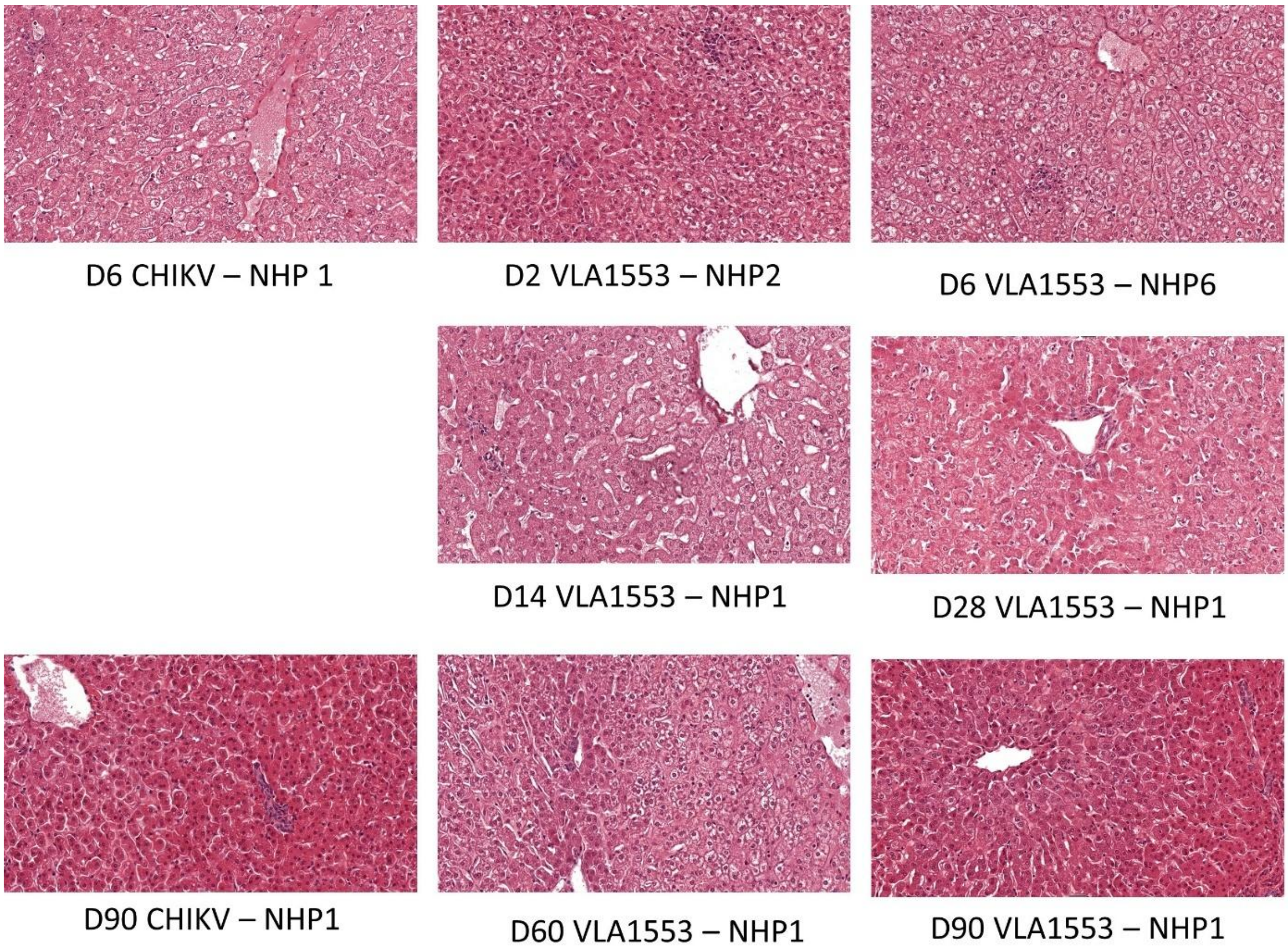

Liver from VLA1553 exposed animals was examined on Days 2, 6, 14, 28, 60, and 90 and compared to liver from WT CHIKV exposed animals from Day 6 and Day 90. Minimal lymphocytic infiltrates were observed in WT exposed animal at day 90 in addition with rare images of hepatocyte dissociation. These infiltrates were not observed in the VLA1553 exposed animals.
